# The Oncobiome in Gastroenteric and Genitourinary Cancers

**DOI:** 10.3390/ijms23179664

**Published:** 2022-08-26

**Authors:** Domenica Lucia D’Antonio, Simona Marchetti, Pamela Pignatelli, Adriano Piattelli, Maria Cristina Curia

**Affiliations:** 1 Department of Medical, Oral and Biotechnological Sciences, “Gabriele d’Annunzio” University of Chieti, 66013 Chieti, Italy; 2 Department of Oral and Maxillofacial Sciences, “Sapienza” University of Rome, 00185 Rome, Italy; 3 School of Dentistry, Saint Camillus International University for Health Sciences (Unicamillus), 00131 Rome, Italy; 4 Fondazione Villaserena per la Ricerca, Città Sant’Angelo, 65013 Pescara, Italy; 5 Casa di Cura Villa Serena, Città Sant’Angelo, 65013 Pescara, Italy

**Keywords:** microbiome, oncobiome, dysbiosis, cancer, tumor-site, gastroenteric genitourinary

## Abstract

Early evidence suggests a strong association of microorganisms with several human cancers, and great efforts have been made to understand the pathophysiology underlying microbial carcinogenesis. Bacterial dysbiosis causes epithelial barrier failure, immune dysregulation and/or genotoxicity and, consequently, creates a tumor-permissive microenvironment. The majority of the bacteria in our body reside in the gastrointestinal tract, known as gut microbiota, which represents a complex and delicate ecosystem. Gut microbes can reach the pancreas, stomach and colon via the bloodstream. Oral bacterial translocations can also occur. In the stomach, pancreas and colon, low microbial diversity is associated with cancer, in particular with a bad prognosis. The urogenital tract also harbors unique microbiota, distinct from the gut microbiota, which might have a role in the urinary and female/male reproductive cancers’ pathogenesis. In healthy women, the majority of bacteria reside in the vagina and cervix and unlike other mucosal sites, the vaginal microbiota exhibits low microbial diversity. Genital dysbiosis might have an active role in the development and/or progression of gynecological malignancies through mechanisms including modulation of oestrogen metabolism. Urinary dysbiosis may influence the pathogenesis of bladder cancer and prostate cancer in males. Modulation of the microbiome via pre, pro and postbiotics, fecal or vaginal microbiota transplantation and engineering bacteria might prove useful in improving cancer treatment response and quality of life. Elucidating the complex host-microbiome interactions will result in prevention and therapeutic efficacy interventions.

## 1. Microbiome and Oncogenesis

The microbiome, often referred to as the “forgotten organ”, comprises all the genetic material within a microbiota that represents ten times that of our cells [1].

When the community of microbes is present in a particular environment, it is referred to as a microbiota, while the set of microbes with their genomes and the surrounding environment is referred to as a microbiome. The microbiota includes various microorganisms such as bacteria, viruses, protozoa, fungi and archaea. This ecosystem is personalized in each individual’s organ, creating a commensal, symbiotic and pathological relationship. Recently, a new focus has been discovered in genomic research called oncobiome. The oncobiome represents the link between the human microbiome and the carcinogenesis process [2]. The International Agency for Research on Cancer estimates that one in five cancer cases worldwide is caused by an infection.

The microbiota preserves the balance in the host and maintains eubiosis, protecting the pathological colonization of the microorganism and cooperating with the metabolic process through a symbiotic agreement. On the other hand, the human intestinal epithelia provide a nutrient-rich microenvironment that tolerates microbiota and an immune system that watches over the invasion of pathogens. Dysbiosis is a change in the normal composition of the microbiome due to an imbalance in the relationship between the host epithelia and the microbiota that can initiate chronic inflammation, epithelial barrier dysfunction and overgrowth of harmful bacteria [3]. These changes in intratumoral or neighboring microbial communities in cancer patients are referred to as the tumor-associated microbiome [4]. Tissue adjacent to tumors is likely to be altered compared to healthy tissue due to factors such as immune cell infiltration, fibrosis and tumor-associated inflammation [5,6,7]. The tissue adjacent to the tumor is in fact similar to tumor tissue in its microbial composition, suggesting a complex interaction between proteins and receptors on the tumor and the surrounding tissue [5]. Many natural and unnatural conditions promote dysbiosis, such as aging, genetic defects, pathogenic microorganisms, transient commensals, antibiotics, xenobiotics, smoking, hormones and dietary cues. All of these well-established risk factors promote inflammatory states that increase the risk of oncogenesis. Numerous microbial species have promoted tumor growth associated with local inflammation [8,9]. The most recognized link between bacteria and non-cardiac gastric cancer is *Helicobacter pylori*. However, elimination of *Helicobacter pylori* has been shown to offer a minimal reduction in gastric cancer, so the evidence that a single organism is the sole cause of cancer remains unlikely while strengthening the evidence for the microbiota.

Inflammation influences the production of specific metabolites such as nitrate. and these allow facultative anaerobic bacteria (e.g., *Enterobacteriaceae*) to grow in a community dominated by obligate anaerobic bacteria lacking the electron transport chain [10,11]. Furthermore, inflammation induces the expression of stress response genes in bacteria, which promotes bacterial fitness and adaptability [12].

The anatomical separation of microbes from the host compartment, which allows symbiotic coexistence, is maintained by multi-level barriers (skin, gut, stomach, pancreas), which are also enriched in immune cells. Barrier defects, due to mutations in genes encoding proteins essential for its integrity and functioning or to infections, inflammation [(absence of key components of inflammasomes as nucleotide-binding oligomerization domain-containing2 (NOD2) and NOD, LRR and pyrin domain-containing6 (NLRP6), or of interleukin-10 (IL-10)], lead to dysbiosis and bacterial translocation and finally have been associated with microbial carcinogenesis.

The role of genotoxins and pro-tumor metabolites released by bacteria is fundamental in carcinogenesis. These influence carcinogenesis by causing DNA damage or negatively affecting the cell cycle. Examples of genotoxins are colibactin, produced by *Escherichia coli*, or P-cresol sulfate (PCS) [13,14].

Accumulated evidence indicates that molecular patterns associated with microorganisms (MAMPs) such as lipopolysaccharide (LPS) and lipoic acid (surface components of gram-negative and gram-positive bacteria) and toll-like receptors (TLRs) interact with each other with pro-inflammatory action to then contribute to carcinogenesis by creating a “microbiota-cancer axis”. These molecules are capable of detecting structures associated with pathogens. In particular, TLR4, the receptor for LPS, promotes carcinogenesis in the colon, liver, pancreas and skin in Tlr4-deficient mice [15,16]. MAMPs are recognized by TLRs and the consequence is the release of reactive oxygen and nitrogen species (ROS and RNS), which could cause DNA damage and mutations [17]. Following pathogen infection, the innate immune system is alerted and is activated by the recognition of ‘non-self’ from ‘self’ through pattern-recognition receptors (PRRs). PRRs, through the recognition of pathogen-associated molecular patterns (PAMPs) or damage-associated molecular patterns (DAMPs), induce interferons (IFNs) of type I and III to enhance junctional barrier function [18]. An intracellular signaling cascade and the upregulation of transcription factors such as NF-κB are caused, which in turn induce IFNs.

PRRs can not only control the microbiota through antibacterial mediators and thereby suppress cancer, but they can also promote resistance to cell death and trigger cancer-promoting inflammation [19]. Several studies report that the microbiome acts remotely to influence sterile tumor environments by influencing both natural autoimmunity and immunomodulating anticancer therapies [20,21,22]. The innate immune system shares many similarities with tumor suppressor signaling, as both processes initiate cell cycle arrest and early apoptotic pathways. However, evasion of innate immunity plays a fundamental role in tumorigenesis. Systemic effects affecting T cells were likely mediated by cross-reactivity between microbial and tumor antigens.

The human microbiome continuously changes throughout the lifespan. Beyond harmless changes, dysbiosis is inherently favored in aging. With increasing age, the microbiota shifts toward a more pro-inflammatory profile that may be linked to adverse health issues, even tumorigenesis in the elderly host. The harmful increase in pro-inflammatory commensals in the gut microbiota can be a primary cause of aging-associated pathologies such as cancer [23]. Microbial dysbiosis has been reported as a feature of aging and it can interplay within the diseasome of aging, modulating several age-related processes, such as genomic methylation levels, low-grade persistent inflammation and diminished nuclear factor erythroid 2–related factor 2 (Nrf2) activity [24].

Of particular interest, Nrf2 is known to decline with age and it plays a crucial role in the fine balance between cell death and survival as follows: it guarantees an anti-tumor function at the physiological level, but it switches to a pro-tumor role when its signaling is exacerbated [25]. In the perspective of cancer being an age-related disease, the key role of Nrf2 in DNA repair and in processing harmful xenobiotics, thereby preventing cancer initiation, raises the possibly important therapeutical role of inducing Nrf2 for treating age-related cancers [26]. From a clinical point of view, it should be crucial to avoid the overexpression of Nrf2, which can favor the progression of cancers such as those of the genitourinary tract [27].

Associated with the transcriptional target heme-oxygenase 1 (HO-1), the Nrf2/HO-1 system plays a protective role under physiological conditions against gastrointestinal cancers as an important protector of the intestinal mucosa [28]. Linking with nutrition, a synergistic interaction between dietary poliphenols and gut microbiota importantly determines the benefits of dietary interventions, also upregulating Nrf2 activity [29].

Within the framework of aging, microbiota and cancer, in addition to Nrf2, it is worth mentioning the signaling of polyamines. Decreases of intracellular and circulating polyamines occur with aging, and polyamine dysregulation is implicated in cancer. Polyamines such as putrescine and spermidine in the intestinal lumen are mainly synthesized by the colonic microbiota. As for Nrf2, the overexpression of polyamines has a detrimental role, e.g., favoring the tumorigenesis of colorectal cancer. In this view, the modulation of polyamine production by gut microbiota may serve as a possible therapeutic target [30].

As an example of the interplay between the genitourinary and gastroenteric microbiome with the aging-associated cancer biology, it has been reported that inflammaging—as the chronic, sterile, low-grade inflammation during aging [31]—and gut and urinary dysbiosis all contribute to immune dysregulation and tumorigenic effects in bladder cancer. Therefore, a therapeutic intervention targeting gut and urinary dysbiosis may deserve further attention in combating bladder cancer, whose incidence, morbidity and mortality are increased with aging [32].

The aim of this review is to examine the intratumoral microbiome and document changes in the commensal microbiota of cancer patients, establishing a correlation with gastric, pancreatic and colorectal carcinogenesis, but also with gynecological cancers, as well as with carcinogenesis of the prostate and the bladder.

## 2. Organ Specific Cancers Microbiome

### 2.1. Gastroenteric Cancers

In the gastroenteric tract, the bacterial community varies between luminal and mucosal-associated communities.

One of the most recognized links between bacteria and cancer is the case of *Helicobacter pylori* in gastric cancer. In contrast to its promotion of gastric carcinogenesis, *Helicobacter pylori* infection reduces the risk of esophageal adenocarcinoma in humans [33], which emphasizes the organ-specific effects of the bacterial microbiota in carcinogenesis. Gastric cancer (GC) is one of the most common cancers in the world and is the first example of carcinogenesis caused by an infection with a specific bacterial pathogen [34]. Among the various risk factors, *Helicobacter pylori* infection plays an important role. *Helicobacter pylori* causes inflammation of the gastric mucosa and a condition of hypochloremia following the destruction of the glands that secrete hydrochloric acid, causing the onset of atrophic gastritis capable of evolving into gastric cancer. In recent years, the world of research has increasingly focused on studying the microbiota and its contribution to the onset of various diseases, such as cancer. In 2006, an interesting study was conducted on the bacteria that characterize the gastric microbiota. Through PCR examinations of biopsy samples and sequence analysis, Bik et al. identified 128 bacterial philotypes [35]. This study revealed that the stomach has a more robust microbiota than previously believed. Subsequently, several studies have focused on the differences in the diversity of the intestinal microbiota as the severity of the phenotype increases, starting from normal gastric mucosa and arriving at the GC situation. In the study by Aviles-Jimenez et al., tissues from patients with superficial gastritis, intestinal metaplasia and gastric cancer were analyzed. The study showed a reduction in gastric microbiota diversity in tumor tissues [36]. However, other studies have shown the opposite, reporting greater diversity of the gastric microbiota in the gastric tissues of cancer patients [37]. Although there is no firm notion of the correlation between gastric microbiota and GC, several studies show that it is plausible that increased or decreased microbiota diversity may be associated with the development of gastric cancer. In particular, there appear to be associations between specific microbes and gastric cancer. For example, the study conducted by Castano-Rodriguez et al. showed a high concentration of *Lactococcus* and *Lactobacillus* in patients with GC. Researchers hypothesized that the mechanism of action underlying the onset of GC is lactic acid production, which may aid tumor progression as lactate acts as an energy source for tumor growth and angiogenesis [38]. A role favoring the onset of GC seems to have several members of the phylum *Nitrospirae* that play a role in the metabolism of nitrates and nitrites [11]. Oral bacteria have been found in patients with gastric cancer, such as *Fusobacterium*, a pro-inflammatory oral bacterial genus [39]. Finally, the production of short-chain fatty acids by *Propionibacterium acnes* seems to contribute to lymphocytic gastritis [40].

A different example showed a decrease in the concentration of *Sphingobium yanoikuyae* in patients with GC. These species degrade aromatic hydrocarbons, a group of molecules with potential carcinogenic effects [41]. Intestinal dysbiosis also plays a role in GC. The researchers studied gastrointestinal hormones on inflammation and gut microbiota in Chinese patients with GC and noted that serum levels of gastrin-17, pepsinogen II, IL-6 and IL-17 are increased in patients with GC and are related to disease severity. The researchers studied the gut microbiota of fecal ampoules before and after chemotherapy and surgery [42]. Treatment with FOLFOX4, a combination of leucovorin (LV) and fluorouracil (FU) with oxaliplatin, restored the optimal intestinal values and surgery led to an increased abundance of *Akkermansia*, *Escherichia/Shigella*, *Lactobacillus* and *Dialister*.

These data are from retrospective studies and are not suitable for establishing a cause-and-effect relationship between dysbiosis and GC. Longitudinal and prospective studies are needed to evaluate changes in the gut microbiota over time and to understand the real cause-and-effect correlation between microbiota and the onset of gastric cancer.

Pancreatic cancer (PC) is one of the malignancies with an infaust prognosis. In addition to common risk factors, several studies have shown the involvement of the microbiota in the onset of PC [43]. Historically, the pancreas was thought to be a sterile organ [44], but recent studies have found the existence of bacteria populations in normal pancreatic tissue and pancreatic ductal adenocarcinoma (PDAC) samples. When comparing patients with PDAC to healthy people, variations in oral, intestinal and intrapancreatic microbiota were noted [45]; furthermore, studies have uncovered the key role of microbes in pancreatic carcinogenesis as well as their influence in modulating the activity of chemotherapies and immunotherapies used for numerous malignancies [46]. Besides the environmental and genetic risk factors, an increasing number of recent reports are showing an association between the composition of the human microbiome and PDAC. Most of the bacterial communities found in the tumoral milieu are present commonly in the gut microbiome [47], suggesting that potentially bacterial translocation from the gut to the pancreas might be occurring. It is demonstrated for the first time in human PDAC patients that the gut microbiota has the capacity to colonize pancreatic tumors and that this colonization can modify the overall microbiome of the tumor. Gut microbes can reach the pancreas through the circulatory system or the biliary/pancreatic duct (transductal transmission) [48], which would demonstrate their potential etiological role in pancreatic cancer. Depletion of the gut microbiota via oral antibiotics restrained tumor growth and metastatic burden in PDAC mouse models [49]. In a study by Ren et al., fecal samples from PDAC patients and matched healthy controls were collected prospectively and analyzed for their microbial characteristics [50]. The gut microbial diversity was found to be significantly lower in PDAC patients. The composition of the gut microbiome was also unique in PDAC patients and contained significantly higher *Bacteroidetes* and lower *Firmicutes* and *Proteobacteria* when compared to healthy controls.

Moreover, some oral bacteria have been shown to confer augmented susceptibility to this neoplasm. In particular, *Porphyromonas gingivalis*, a gram-negative anaerobic pathogen, has been linked to a high risk of developing pancreatic cancer. Lu and colleagues found that the microbiome diversity of the tongue coat in PDAC patients was significantly increased, and the bacterial composition was markedly different from controls [51]. A few bacterial genera (*Haemophilus*, *Porphyromonas*, *Leptotrichia* and *Fusobacterium*) could distinguish PDAC patients from healthy individuals [52]. Interestingly, periodontal disease has been linked to increased PDAC risk that may be related to oral dysbiosis. *Porphyromonas gingivalis* is an important contributor to periodontal disease and may cause systemic inflammation, leading to carcinogenesis. Results from some studies showed that higher levels of antibodies against a pathogenic strain of *Porphyromonas gingivalis* were associated with a two-fold increase in the risk of PDAC. While higher levels of antibodies against commensal oral bacteria were associated with a lower risk of PDAC [53]. In a recent study, the authors found that long-term survivors (five years or more) have higher intratumoral microbiome diversity as compared to short-term survivors, demonstrating that intratumoral microbiome composition can be an indicator of PDAC patients’ survival. LTS showed enrichment of *Proteobacteria* (*Pseudoxanthomonas*) and *Actinobacteria* (*Saccharopolyspora* and *Streptomyces*), while no predominant genus was detected in STS tumors. The tumor microbiome diversity has a powerful impact on determining the survival of PDAC patients. The microbiome unique to LTS may contribute towards shaping a favorable tumor microenvironment, characterized by the recruitment and activation of CD8 T cells to the tumor milieu and it might also be useful as a predictor of patients’ outcomes. Another key consideration is the role of the microbiome on the immune system. Erick Riquelme et al. found higher CD3+ and CD8 + T cell densities in LTS than in STS patients, as well as significantly higher numbers of Granzyme B+ (GzmB) cells in LTS [54]. An important factor that can cause dysbiosis is metabolic dysfunction [55]. For this reason, the influence of metabolic processes on the intratumoral microbiome has been examined. If in LTS cases there is an enrichment of the pathways linked to the metabolism of amino acids, xenobiotics, lipids, terpenoids and polyketides, the cases of STS have shown an enrichment in the synthesis and processing of proteins, in the processing of genetic information, in energy and nucleotide metabolism, in replication and repair. Furthermore, bacteria could influence immune infiltration, which ultimately affects PDAC survival.

It has long been suspected that the alterations in the microbiota may influence colorectal carcinogenesis. CRC is known to be essentially a genetic disease, but it is still unknown precisely what events contribute to precipitating the initial disease and promoting progression.

It is thought that the bacterium acts similar to a “hit and run” in the sense that even limited exposure to the bacterium is sufficient to incite the disease, even when the tumor microenvironment is no longer hospitable for the life of the bacterium [56]. It is also possible that the characteristics of transformed colonic epithelial cells render them more sensitive to microbially-influenced carcinogenesis. Driver gene mutations give epithelial cells the ability to requisition immune cells to further promote growth and spread, but the bacteria behave as a network of genes that influence the stability of the genome, the metabolism and the immune response.

Furthermore, the interactions between microbiota and hosts are influenced by host genetic polymorphisms that modify immune and metabolic responses. Furthermore, a bacterial biofilm on the epithelial cell first creates an inflammatory microenvironment due to the initial production of cytokines and ROS, which then transforms into a tumor microenvironment [57]. The colon appears to be the organ most subject to developing cancer and the part of the digestive tract with the highest microbial concentration [58]. The microbiota that characterized the CRC is richer in some bacterial pro-inflammatory species (*Streptococcus gallolyticus*, *Fusobacterium nucleatum*, *Escherichia coli*, *Bacteroides fragilis* and *Enterococcus faecalis*) and more depleted in butyrate-producing bacterial species (*Roseburia*, *Clostridium*, *Faecalibacterium* and *Bifidobacterium*) [59,60]. Although there is significant interest in identifying specific oncomicrobes, no single species has been found to be universally present among all individuals with CRC and there is significant variation in microbial composition between individuals [56]. In the CRC, there is not a specific microorganism responsible for the onset of the tumor but a group of bacteria that may act synergistically whose harmful actions exceed those of the benefits of the resident commensals. Interestingly, microbiome alterations also occur with colorectal adenoma, the early stage of CRC. As in tumors treated so far, microbial diversity is also reduced in CRC patients compared to healthy controls [39]. In particular, *Fusobacterium nucleatum* and *Actinobacteria* are among the most enriched taxa in CRC patients [61]. Besides these, *Peptostreptococcus*, *Prevotella*, *Parvimonas* and *Twin* can also be effective biomarkers for detecting CRC [62]. In a recent study, a significantly increased concentration of *Fusobacterium nucleatum* was observed in patients with early-stage CRC, indicating a worse prognosis [6]. Despite the variations in intestinal microbiota, several individual bacterial species have been associated with CRC. *Streptococcus bovis*, a gram-positive cocci, is a reported risk factor for CRC. *Enterotoxigenic Bacteroides fragilis*, a bacterium producing *Bacteroides fragilis* toxin (BFT), causes diarrhea and inflammatory bowel disease (IBD) [63]. Some studies reported that *Enterococcus faecalis* was significantly higher in patients with CRC compared with that in healthy controls. *Enterococcus faecalis* infection induces superoxide production, which damages DNA in epithelial cells [64]. Although *Escherichia coli* is a gut commensal bacterium, studies have reported higher levels of colonic colonization by mucosa-associated *Escherichia coli* in CRC patients compared with that in healthy people [65]. It is postulated that the mechanism underlying the correlation between microbiota and CRC is the Driver-Passenger Model [57]. In this model, bacteria are divided into the following two groups: in the early stages of CRC, there are bacteria called “drivers” that produce genotoxic substances to damage the DNA of epithelial cells. In the advanced stages of CRC, there are “passenger” opportunistic bacteria. In this model, oral microbes such as *Fusobacterium nucleatum* colonize the gut epithelial cells and act as a bridge for other secondary oral microbes (*Porphyromonas* spp., *Peptostreptococcus* spp. and *Parvimonas* spp.) by means of adhesins. The oral microbes form a biofilm that alters tight epithelial junctions and promotes inflammation by mucosal immune cells.

*Fusobacterium nucleatum* and *Porphyromonas* can invade epithelial cells, disrupting signaling and promoting transformation. The transformation of epithelial cells leads to an oncogenic synergy in which host-secreted peptides feed the oral asaccharolytic microbes, which in turn produce reactive oxygen species (ROS). At this point, both continued biofilm formation and inflammatory responses are promoted as the tumor grows. *Fusobacterium nucleatum* first generates a pro-inflammatory microenvironment by recruiting immune cells infiltrating the tumor (TIL), then down-regulates the adaptive anti-tumor immune response mediated by T lymphocytes, creating a tumor microenvironment. The breakdown of the intestinal barrier appears to be a major cause of CRC. The intestinal epithelial cells (IEC) form a physical barrier that separates the intestinal microbiota from the internal intestinal tissue with the function of forming effective protection from the external environment and from the invasion of bacteria [66]. Following inflammation, colon epithelial cells become unable to form this barrier, allowing bacteria to invade and induce tumorigenesis [67]. The “driver” *Fusobacterium nucleatum* bacterium adheres to the intestinal epithelial cell membrane through its adhesin A (FadA), which selectively binds to E-cadherin and activates the β-catenin signaling pathway, thus inducing oncogenic and inflammatory responses [68]. Furthermore, its surface adhesin Fap2 induces the secretion of proinflammatory cytokines, IL-8 and CXCL1, which promote CRC cell migration [69]. A later published study by the same authors explained in greater detail the oncogenetic role of *Fusobacterium nucleatum* in colorectal carcinogenesis and in particular in the Wnt/β-catenin signaling [70]. *Fusobacterium nucleatum* is able to bind to Annexin A1, a previously unrecognized modulator of Wnt/β-catenin signaling. Proliferating colorectal cancer cells show an increased expression of Annexin A1, which in turn enhances *Fusobacterium nucleatum* binding, forming a more stable complex. The authors speculated that the increased expression of Annexin A1 is the first “hit” and microbes such as *Fusobacterium nucleatum* are the second “hit” to aggravate cancer progression. This model identifies microbes as facilitators for CRC [70].

The intestinal tract is the largest mucosal surface of the human body. It has a critical role in protecting the host from the environment while maintaining proper nutrient absorption. Normally, the intestinal mucosal barrier isolates the intestinal microbiota from immune cells. The intestinal mucosal barrier is lined by a single layer of IECs joined by tight junctions [71], which forms a barrier between the intestinal lumen and the host’s lamina propria. The intestinal mucosal barrier is highly permeable. All these suggest that transformed IECs fail to form an effective surface barrier, enabling commensal bacteria and their degradation products to invade the tumor stoma. The host recognizes the microbiota via various pattern recognition receptors [PRRs, such as Toll-like receptors (TLRs)], which control the inflammatory response to microorganism-associated molecular patterns, such as lipopolysaccharide. Metabolites produced by the intestinal microbiota also appear to be involved in the process of inflammation and carcinogenesis, such as butyrate or tryptophan, protecting from the onset of CRC [72].

One of the best-characterized examples of microbial host interactions is the bidirectional interaction called the brain-gut-microbiome (BGM) axis between microbes, enterochromaffin cells (ECCs) and the central nervous system (CNS) [73]. Data obtained in human beings suggests that alterations in these interactions may play a role in several brain-gut disorders. The following three are the main components of the BGM axis: the CNS, the autonomic nervous system (ANS) and the gut microbiota. Growing evidence has sought to unravel the intricate balance between them and to shed light on the involvement of the axis in tumor genesis, proliferation and growth. Colorectal cancers are believed to be the most important and extensive representation of the BGM axis [74]. Under normal conditions, the communication from the gut to the CNS is autonomous. The ANS regulates gut functions, including motility, antimicrobial peptide production, intestinal permeability and the mucosal immune response. These changes affect the microbial habitat, thereby modulating the composition and activity of the microbiota. On the contrary, in pathological conditions, signals may reach the somatic sensory system and lead to gastrointestinal dysfunction. Gut microbes and their metabolites communicate with the CNS by different pathways, including the neuroendocrine and enteroendocrine signaling pathways, involving the vagus nerve, the enteroendocrine cells (EECs), cytokines and neurotransmitters [75]. This communication is mediated by several microbially derived molecules that include short-chain fatty acids (SCFAs) and tryptophan metabolites [76]. These molecules propagate signals through interaction with the mucosal immune system and ECCs, which in the gut wall function as an interface between the organism and the gut lumen. They may also cross the intestinal barrier, migrate to other parts of the body via the circulatory system, cross the blood-brain barrier and cause secretion of various neuroactive molecules, thus affecting inflammation and tumorigenesis in specific organs [77,78].

In addition to generating these CNS-activating metabolites, the microbiota can produce neuroactive molecules including γ-aminobutyric acid (GABA), 5-HT and dopamine, although it is not known if in enough levels to elicit a host response. The ANS can activate ECC to produce and release 5-HT into the gut lumen where it interacts with gut microbes [79]. Considering the central role of 5-HT in regulating gastrointestinal motility and secretion, there is an immense selective pressure on gut microbes to act on the serotonergic system. The essential amino acid tryptophan (Trp) is the precursor to the neurotransmitter 5-HT and, thus, it represents a key molecule in the BGM [80]. Dietary intake of proteins that contain Trp and the action of the intestinal microbiota on its peripheral availability are the main regulators of the peripheral availability of the amino acid since the host is unable to produce it. Gastrointestinal tract endocrine cells can produce upwards of twenty different hormones, which can have an effect on the microbiota closely located in the gastrointestinal mucosa. The gut hormones act together with immune mediators in the communication between the brain and the microbiota.

Many gastrointestinal tumors can be infiltrated and innervated by nerves [81]. This perineural tumor invasion is important because it has prognostic value. Nerve cells involved in perineural invasion can secrete neurotransmitters or neuropeptides, which include serotonin, 5HT and GABA, which play a role in modulating tumor proliferation, migration, invasion and angiogenesis [82]. Preclinical evidence has shown that abnormal bidirectional interactions within the BMG axis can result in gastrointestinal diseases, such as Inflammatory Bowel Syndrome [83].

Normally there is a balance between protective, butyrate-producing populations and inflammatory, mucin-degrading populations, but with age the microbiota changes. There is therefore a reduction in butyrate production and an increase in the intra-colonic pH values, creating a hostile environment for colonocytes. The pH rises from the cecum to the rectum, and that provides a plausible explanation for the growing susceptibility to tumorigenesis in these intestine sites [84].

**Figure 1:**
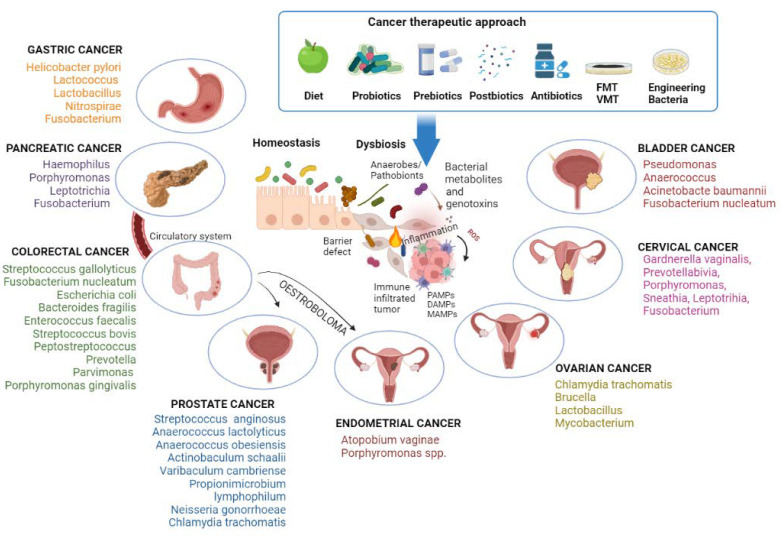
The condition of dysbiosis, mainly the increase in anaerobes/pathobionts, promotes carcinogenesis. Bacterial genotoxins damage cellular DNA, stimulate local inflammation, and activate the innate immune system through the molecular patterns associated with microorganisms (MAMPs), the pathogen-associated molecular patterns (PAMPs), or the damage-associated molecular patterns (DAMPs). Gut pathogenic bacteria can directly drive the process of pancreatic carcinogenesis by migrating through the bloodstream or indirectly influence endometrial and prostate carcinogenesis through oestrobolome. Different anticancer therapies act by counteracting dysbiosis. Image created with BioRender (https://biorender.com; accessed on 1 July 2022). FMT: transplantation of fecal microbiota; VMT: transplantation of vaginal microbiota.

An overview of the gastroenteric cancer-associated microbiome is shown in Figure 1.

### 2.2. Genitourinary Cancers

#### 2.2.1. Female Reproductive Tract (FTR)

The FRT microbiota interacts with the gut and with the urinary tract, defining a vagina–gut axis and a vagina–bladder axis, respectively [85,86]. FRT also influences other distal mucosal sites, for example, the oral cavity, directly or through mechanisms mediated by oestrogens. Enteric bacteria metabolize circulating oestrogens and the set of these microorganisms, and their genes is termed the *oestrobolome* [87]. Thus, a lack of oestrogen-metabolizing bacteria leading to a reduction in the gut microbiota diversity could influence the vaginal microbiome composition.

Anatomically, the FRT can be divided into the lower (vagina and cervix) and upper (endometrium, Fallopian tubes and ovaries) FRT. In healthy women of reproductive age, the vaginal microbiota mostly exhibits low microbial diversity (defined as species richness and evenness) consisting of a few *Lactobacillus* spp. (*crispatus*, *gasseri*, *iners*, *jensenii* or *vaginalis*) [88,89,90]. This characteristic is in contrast to other mucosal sites, for instance, the colon, in which this situation is considered pathologic. The dominance of *Lactobacillus crispatus* may be optimal for vaginal health, while the dominance of *Lactobacillus iners* may be less beneficial. The bacteria residing in the lower FRT, including *Lactobacillus species* and dysbiotic anaerobes, can ascend to the upper FRT and can colonize the rectum. [90]. In return, the vaginal epithelium secretes glycogen and the high levels of free glycogen promote the growth of *Lactobacillus* spp., which use glycogen breakdown products as an energy source through fermentation [91]. As a consequence of this metabolic process, *Lactobacillus* spp. produces lactic acid, which protects the vaginal microenvironment. Due to the changes in oestrogen levels, the vaginal microbiome is a dynamic ecosystem that takes into account the phases of the menstrual cycle. Lactic acid produced by predominant vaginal *Lactobacillus* spp. protects the environment of the vagina from invading pathogens by maintaining the low pH of the cervicovaginal region [89,92]. This creates a mutually beneficial relationship. These microorganisms also produce bacteriocins that block the adhesion of incursive pathogens to the vaginal epithelium [88,89,92,93,94]. Numerous in vitro studies have attributed the beneficial effect of *Lactobacillus* spp. to hydrogen peroxide. Protonated lactic acid kills sexually transmitted pathogens, including *Neisseria gonorrhoeae* [95], *Chlamydia trachomatis* [96], *herpes simplex virus* [97] and *human immunodeficiency virus* [98], as well as uropathogenic *Escherichia coli* [99].

Hematogenous spread of bacteria emanating from the distal mucosal sites, such as the gut or oral cavity, occurring during epithelial barrier breach (e.g., gingivitis and leaky gut) [100,101] might be a putative seeding route for the upper FRT microbiome [85].

Multiple factors have been shown to influence the vaginal microbiome. These can be behavioral (sexual orientation, sexual activity, number of sexual partners, use of sexual lubricants, contraception, feminine hygiene practices, smoking and vaping, alcohol consumption, diet and/or nutrition, obesity and physical activity), socioeconomic (e0ducation, income, structured racism and/or segregation, social policies and acce10ss to healthcare), genetic or host-related (age, genome and epigenome, hormonal status, pregnancy and impaired immunity), other comorbidities (cardiometabolic, neuroendocrine and immunoinflammatory) and environmental (sexually transmitted disease status, *Human Papilloma Virus* (*HPV*) vaccination, stress, antibiotics, probiotics, xenobiotics, toxins, carcinogens, geography and early childhood factors such as gestation, birth and childhood path) [89,92,93,102,103]. Estrogen levels, in particular, have a profound effect on the composition of the vaginal microbiome. For example, before puberty or in postmenopausal women, when circulating estrogen levels are low, the vaginal microbiome is devoid of *Lactobacillus* spp. and is made up of a diverse mix of anaerobic bacteria [104]. Conversely, the vaginal microbiomes of pregnant women, which are prone to high levels of estrogen, are more stable and typically dominated by *Lactobacillus crispatus* or *Lactobacillus iners* [105]. Longitudinal studies have revealed that the vaginal microbiome is a dynamic ecosystem, which can fluctuate over short periods of time in some women or be relatively stable in others [106,107,108]. In particular, it has been shown that higher ratios of l-lactic acid to d-lactic acid in predominantly *Lactobacillus iners* communities or in several non-*Lactobacillus* predominantly communities are correlated with elevated levels of extracellular matrix metalloproteinase inducer and, consequently, matrix metalloproteinase 8 (MMP8) in vaginal secretions, which could alter the integrity of the epithelial barrier in these women [108]. The vaginal microbiome may also present pathogens, such as *Streptococcus* spp., *Staphylococcus* spp. or *Enterobacteriaceae* [109], which have been associated with numerous gynecological and obstetric diseases, including pelvic inflammatory disease, endometritis and gynecological cancer [89]. The relative abundance of *Lactobacillus* gradually decreases throughout the upper FRT, with the lowest abundance in the fallopian tubes [110]. The upper FRT microbiota differs considerably from that of the vagina in both quantity and composition, with a bacterial load reduced by 10,000 times. Unlike the microbiota of the vagina, in which the upper FRT presents greater bacterial diversity [110], it is unclear if these bacterial species are resident or transient colonizers coming from the lower FRT. Moreover, the residing microbiota is not well characterized yet.

Gynecologic cancers, which begin in the reproductive organs of females, commonly include cancer of the cervix, endometrium and ovary. Cancers of the vagina are rare, but most of the time, bacteria that colonize the vagina are responsible for cervical cancers (low FRT) and endometrium, ovary and fallopian tubes (upper FRT). The vaginal microbiome may travel and colonize other organs (e.g., skin, lungs, bladder, prostate gland and urethra). Cancers of the vulva are also rare.

The gut and vaginal microbiota produce metabolites like endotoxins, bile acids, lipopolysaccharides, genotoxins and conjugated estrogen, which induce DNA damage and increase genomic instability. These metabolites may activate oncogenic signaling locally as well as distantly, for example in the breast and in FRT [111,112,113,114,115].

Cervical cancer is the most common *HPV*-related malignancy, but it is possible that factors in the local cervicovaginal microenvironment, such as the vaginal microbiome, might promote carcinogenesis in conjunction with *Human Papilloma Virus*. The exact mechanism of interaction between the *Human Papilloma Virus* and vaginal bacteria has not yet been identified, although an attempt has been made in an in vitro study on cell lines and organoids [116]. During cervical carcinogenesis, elevated anti-inflammatory IL-4 and transforming growth factor β1 (TGFβ1) have been associated with the presence of *Fusobacterium* spp., indicating its effect on host response and in particular, its involvement in the development of the immunosuppressive microenvironment [117]. The rapid decline in estrogen concentration in menopause is associated with a decrease in *Lactobacilli* composition. This increases the alkalinity of the cervico-vaginal environment, leading to the abundance of other anaerobic bacteria such as *Gardnerella vaginalis*, *Prevotellabivia*, *Porphyromonas*, *Sneathia*, *Leptotrihia* and *Fusobacterium,* rendering the cervical cells susceptible to oncogenesis [118,119,120].

Known risk factors for the development of endometrial cancer [121] are environmental factors, including obesity, inflammation, imbalances in oestrogen metabolism and they are also strongly associated with changes in the gut [122] and vagina microbiomes [85,123]. The gut microbiome might indirectly promote endometrial carcinogenesis by altering genital microbial communities. Endometrial cancer is reported to have the presence of *Atopobium vaginae* and *Porphyromonas* species, which colonize the endometrium, first inducing hyperplasia and then carcinoma. Sequencing analysis of *Atopobium* and *Porphyromonas* species found in samples from patients with endometrial cancer showed a high match with bacteria of the same species found in the vagina [124]. The association between endometrial cancer and these bacterial species found in lower FRT has been definitely assessed [125].

Ovarian cancer is one of the most lethal malignancies of the female reproductive system, primarily because of its asymptomatic nature during the early stages of development. Significantly elevated levels of *Proteobacteria* and *Firmicutes* phylum bacteria, with *Chlamydia trachomatis*, *Lactobacillus* and *Mycobacterium,* have been reported in ovarian cancer. *Chlamydia* is known to contribute to cancer by inhibiting apoptosis, inducing DNA damage response and increasing susceptibility to other infections [126]. In addition to the known risk factors for ovarian cancer, such as genetic predisposition, early ovulation and late menopause, nullity, obesity and fertility medication/hormone therapy, an alteration of the microbiota is emerging [127,128]. Multiple reports indicate the association between a comparative decrease in intestinal microbiota diversity and ovarian cancer [129]. The bacteria which normally colonize the ovary are the following: *Chlamydia trachomatis*, *Mycoplasma genitalium*, *Proteobacteria*, *Firmicutes*, *Bacteroides*, *Actinobacteria*, *Chloroflexi*, *Acidobacteria*, *Fusobacterium*, *Acinetobacter*, *Sphingomonas* and *Methylobacterium* along with *HPV*. Early evidence suggests that *Proteobacteria* and *Firmicutes* phylum are significantly increased in ovarian cancer tumors [130]. Furthermore, the presence of *Brucella*, *Mycoplasma* and *Chlamydia* in more than 60% of the screened samples along with *Chlamydia trachomatis*, *Lactobacillus* and *Mycobacterium* species suggests an association of disrupted microbiota composition with the development of ovarian cancer [126,130,131,132,133,134]. The causational link between microbiota and ovarian cancer remains unclear. These microorganisms might induce carcinogenesis through direct or indirect mechanisms; however, the highly anoxic tumor microenvironment might also favor the recruitment and growth of anaerobic microorganisms, such as *Chlamydia* spp. [130]. The study by Banerjee et al. has identified a microbiome signature unique to ovarian cancers, showing that the microbiome of ovarian tumors is quite different from its surrounding non-cancerous tissue and very different from ovarian tissue very distant from the tumor.

#### 2.2.2. Male Reproductive Tract (MTR)

Little is known about the microbiome of the MTR and there are only a few studies on the male urine microbiota (MUM). The healthy MUM is characterized by genera such as *Lactobacillus*, *Sneathia*, *Veillonella*, *Corynebacterium Prevotella*, *Streptococcus* and *Ureaplasma*, and these are also found on urethral swabs [135,136]. A study of men with and without sexually transmitted infections (STIs) found that bacteria associated with STIs but also with vaginal dysbiosis can be found in STI-positive patients [137].

Prostate cancer is the second most typically diagnosed cancer and the sixth leading cause of cancer death worldwide, with an estimated 1,276,000 new cancer cases and 359,000 deaths in 2018 [138]. It is currently known that chronic inflammation is associated with the carcinogenesis process. Among the leading causes of inflammation are infections, hormonal alterations, physical trauma, the breakdown of the epithelial barrier, urinary reflux and diets rich in carcinogens that can reach the prostate and cause DNA damage [139]. The inflammatory state is characterized by an infiltration of immune cells (macrophages, neutrophils and lymphocytes), which release reactive oxygen species (ROS), reactive nitrogen species and pro-inflammatory cytokines, causing DNA damage, cell damage and cell death. The consequent chronic inflammation state promotes epithelial cell regeneration, creating proliferative inflammatory atrophy (PIA), which evolves into prostatic intraepithelial neoplasia (PIN) and finally prostate adenocarcinoma. There is increasing consciousness of the role of the microbiota in stimulating the state of chronic inflammation and its probable involvement in the maturation of prostate cancer [140]. Cavarretta et al. examined the microbial existence in prostate tissue’s tumor, peritumoral and non-tumor areas using 16S rDNA sequencing directed to V3–V5 hypervariable regions. *Propionibacterium* spp. was the most predominant bacterial genus found in all tumor sites, and its presence was particularly associated with prostate tissue inflammation. Beta diversity was not significantly different between sites, but individual bacterial species were mainly and differentially abundant in some areas. Tumor and peritumoral regions had similarly higher relative abundances of *Staphylococcus* spp. than normal areas. In distinction, normal areas had a greater abundance of *Streptococcus* spp. than tumor and peritumoral regions [141]. A study by Feng et al. used integrated metagenomics and metatrascriptomics analysis to define the microbiota in frozen free radical prostate fragments from tumors and adjacent benign tissue of patients. The authors identified over 40 unique bacterial genera, of which the most abundant were *Pseudomonas*, *Escherichia*, *Acinetobacter* and *Propionibacterium* spp. The study found no differences between tumors and benign tissue in terms of overall bacterial diversity (alpha) or group diversity (beta), regardless of the Gleason score [142]. Prostate cancer has been associated with chronic infections of the urinary tract such as chronic prostatitis and chronic pelvic pain syndrome. The finding of a vast urinary microbial diversity changed the previous principles of urine sterility. Thus [143,144], understanding the urinary microbiome is essential in linking the dots in the pathogenesis of prostate cancer. Previously, urine microbiome investigation by culture faced a fundamental challenge of contamination from the skin, foreskin, virginal and rectal areas. Advances have overcome this challenge in new, susceptible detection methods such as 16S RNA and DNA sequencing and shotgun metagenomics sequencing [145]. Several studies have examined the connection between the urinary microbiome and prostate cancer [146,147]. Shrestha and colleagues studied urine samples from men before undergoing prostate biopsy to determine whether the urinary microbiome could be associated with the presence of cancer, the degree of cancer and the type and degree of prostate inflammation [146]. The study revealed that men with prostate cancer had a higher rate of a group of bacteria associated with urogenital infections compared to negative biopsy samples. This cluster consisted of *Streptococcus anginosus*, *Anaerococcus lactolyticus*, *Anaerococcus obesiensis*, *Actinobaculum schaalii*, *Varibaculum cambriense* and *Propionimicrobium lymphophilum*. Some species were present in a different entity in the presence or absence of acute inflammation or in high-grade tumors than in low-grade ones. A prior study estimated the possible correlation between bacteria and prostate cancer by examining the type of microbiota in expressed prostatic secretions (EPS) of patients with prostate cancer and benign prostatic hyperplasia (BPH). The results showed a significant increase in *Bacteroidetes*, *Alphaproteobacteria*, *Firmicutes*, *Lachnospiraceae*, *Propionicimonas*, *Sphingomonas and Ochrobactrum*, *while Eubacterium* and *Defluviicoccus* decreased in the prostate cancer group, compared to the BPH group. In addition, *Escherichia coli* was significantly reduced in the urine of the prostate cancer group, despite an increase in EPS and semen. Enterococcal counts, on the other hand, increased substantially in seminal fluid, with little changes in urine and EPS [148]. In a study by Alanee et al., the urinary microbiota of men undergoing transrectal prostate biopsy with elevated prostate-specific antigen (PSA) levels were evaluated. Urine samples were taken, followed by a prostate massage. Prostate cancer patients had a high abundance of *Veillonella*, *Streptococcus* and *Bacteroides* and a low abundance of *Faecalibacterium*, *Lactobacilli* and *Acinetobacter* compared to those with BPH [149]. Nevertheless, this analysis had a limited sample size of patients, which would have decreased the power of the study. From the results of several studies on the urinary microbiome, it remains critical to standardize procedures and techniques for collecting urine samples. This will furnish a platform for similar results [150]. Prostate cancer’s risk and pathogenesis can also be modulated by the gut microbiome [151], whose composition regulates the metabolism of compounds associated with increased prostate cancer risk. Analyses have revealed that a regular dietary composition of dairy products, red meat and high fat is associated with an improved prostate cancer risk [152]. Antibiotics have been shown to cause gut microbial dysbiosis, which can propagate translocation of pathogenic bacteria, leading to chronic inflammation, an essential inducer of tumorigenesis [153]. Plottel et al., 2011 hypothesized that the *oestrobolome* is associated with prostate cancer risk. Estrogen is said to activate polycyclic hydrocarbons, conducting the formation of carcinogenic metabolites, e.g., radical cations that induce cellular DNA damage, leading to carcinogenesis [65]. Sfanos et al. 2018, carried out a cross-sectional analysis where they profiled the fecal microbiota of healthy male volunteers and men with different clinical states of prostate cancer (i.e., localized, biochemically recurrent and metastatic disease) using 16S rDNA amplicon sequencing. The analysis registered greater alpha diversity in those without prostate cancer than in those with prostate cancer [154]. The results from different studies indicate a plausible link between specific gut microbial species and prostate cancer risk and disease status. Chronic inflammation, coupled with prostate and/or urinary tract infections, provides an inflammatory microenvironment that promotes the development of prostate cancer precursor lesions that drive prostate tumorigenesis [155]. Understanding the microbiota’s potential implications for different prostate cancer elements remains largely underexplored. Whether specific microbiota is causative in prostate cancer, and if so, how, remains to be determined.

The genital microbiota interacts with the urinary tract, probably by translocations of both uropathogens and health-associated bacteria [86]. Sequencing analyses of the microbiome highlighted its interconnection in that the same taxa are found in different but close organs, such as the bladder and vagina in women (bladder-vaginal axis) or the urethra and prostate in men [155,156]. The microbiome is also shared between sexual partners, such as the penile skin, urethra and semen of male partners and the vagina of female partners) [86,157,158]. The urogenital microbiota is a potential risk factor in the development and progression of genitourinary cancers (Figure 1).

#### 2.2.3. Urinary Tract

Although the number of analyses is restricted, some authors found essential distinctions between the urinary microbiota of men and women [156,159]. This outcome is not surprising given the differences in the anatomical system, hormones and local defenses. Ultra-deep pyrosequencing showed that the most abundant bacterial taxa in the urine of healthy individuals are the following: *Lactobacillus*, *Corynebacterium*, *Prevotella*, *Staphylococcus*, *Gardnerella* and *Streptococcus*, with a preponderance of *Prevotella*, *Lactobacillus* and *Gardnerella* in women and *Corynebacterium* in men [156,160]. The urinary tract has its own microbiome because urine passing through the urethra is contiguous to the external environment and exposed to the skin and the openings of the gastrointestinal tract and vaginal mucosae, which host their microbiota. Like intestinal microbiota, urinary microbiota is age-dependent [159], with important distinctions among age groups. Data from studies of midstream urine (used as a proxy of the bladder microbiome) suggested that a more heterogeneous mix of bacterial genera exists in women’s samples (6–36 genera) than in men’s samples (from 1 to 8 genera, but also one sample with 51 genera) [159]. Moreover, regardless of sex, in the majority of samples, more than 50% of bacteria appertained ed to the phylum *Firmicutes*. Women’s samples also had members of the phyla *Actinobacteria* and *Bacteroidetes*, which were generally missing from the male samples. In addition, midstream urinary research proved the presence of a core microbiome, also in the bladder, but with variability in the abundance of the core bacteria, along with a variable preponderance of other bacteria, across age groups. This statement was more valid in the urinary tract than in the gut microbiome [161,162], sustaining the hypothesis that bladder colonization with specific genera throughout a lifetime might impact the disposition for bladder pathology in later life. These findings might also elucidate the distinction in the frequency of urinary diseases observed in men and women.

Bladder cancer remains the most common malignancy of the urinary tract. In 2018, it was diagnosed in 549,393 patients and 199,922 succumbed to the disease worldwide [163]. Emerging data have discredited the documented opinion that the urine and bladder are sterile in healthy individuals [159,164,165,166,167]. Current culture and sequencing techniques have now allowed the detection of microbes throughout the urinary system [147,168]. A growing direction of bladder cancer research is directly aimed at comprehending how the commensal urinary microbiome can impact vulnerability to bladder cancer development and its impact on treatment effectiveness.

The effect of microbes on bladder cancer carcinogenesis is perhaps most clear from the observation that squamous cell carcinoma of the bladder is related to urogenital schistosomiasis [169]. *Schistosoma haematobium* has always been associated with this kind of bladder cancer. Its pathogenic role may arise through several mechanisms, such as epithelium injury, chronic inflammation and oxidative stress [170]. To date, few studies have reported a detailed analysis of the urinary microenvironment of urothelial bladder cancer [171], comparing the urine microbiota of healthy individuals and patients with bladder cancer. Their initial results showed an enrichment of *Streptococcus* in urine from patients with urothelial carcinoma, while *Streptococcus* abundance was near zero in nearly all healthy patients. In cancer samples, *Pseudomonas* or *Anaerococcus* were the most abundant genera, where *Streptococcus* abundance was low. An equivalent study analogized bacterial communities between urine samples of healthy individuals and cancer patients [172]. The authors discovered that the most abundant phylum in both groups was *Firmicutes*, followed by *Actinobacteria, Bacteroidetes* and *Proteobacteria*. They determined operational taxonomic units (OTUs) belonging to the genus *Fusobacterium* to be more abundant in the bladder cancer group. An independent group of bladder cancer tissues was analyzed and confirmed the detection of *Fusobacterium nucleatum* sequences. The genera more abundant in healthy urine were *Veillonella*, *Streptococcus* and *Corynebacterium* [172]. More recently, bladder cancer patients were found to have increased bacterial richness, defined by the number of unique OTUs in a sample [173].

The European Organization for Research and Treatment of Cancer (EORTC) scoring system highlighted an incredible bacterial richness in urine from non-muscle invasive bladder cancer (NMIB) patients with a high risk of recurrence or advancement. Therefore, the authors suggested that in NMIBC, higher bacterial richness may indicate a high risk of progression and recurrence.

*Acinetobacter* and *Anaerococcus* were found in higher abundance in bladder cancer patients compared to the non-cancer group [173]. Virulence factors of *Acinetobacter baumannii* include invasion of epithelial cells, phospholipid degradation and biofilm construction, which encourages escape from the host immune response [174]. Member of the Gram-positive anaerobic cocci, the *Anaerococcus* was documented to induce inflammation and remodeling of extracellular matrix (ECM) [175]. The authors introduce the possibility that the interplay of ECM, microbiome, and inflammation plays a crucial role in bladder cancer onset, advancement, and relapse [176]. In 2002, Seow et al. [177] found that *Lactobacillus casei* and *Lactobacillus rhamnosus GG* inhibited the growth of bladder cancer cells by stimulating a cytotoxic effect. Accordingly, Ohashi et al. [178] performed a case-control matched study to analyze bladder cancer risk reduction associated with the consumption of products based on fermented milk. The probability ratio for recurrence was 0.46 (95% confidence interval: 0.27–0.79) for consuming fermented milk products 1–2 times per week versus less than 1–2 times per month. The outcomes indicated that the regular intake of lactic acid bacteria decreased the risk of bladder cancer. Indeed, epidemiological analyses have demonstrated that UBC incidence depends on age and that men have a more elevated risk than women, with a rate proportion of at least 3:1 [179,180]. The relationship between bladder-associated microbiota and cancer incidence in men and women has not yet been comprehensively assessed. For example, whether the dominant bacterial strain *Lactobacillus* in the bladder of women might protect against UBC is unknown, although many reports have shown that *Lactobacillus* might reduce chronic inflammation and potentiate several immune responses [181,182,183]. A multicenter, double-blind, placebo-controlled, randomized trial in patients with primary bladder tumors documented that daily oral administration of freeze-dried *Lactobacillus casei* sp. *Shirota* for 1 year controlled the recurrence of UBC after transurethral resection of the tumors [184]. Another multicenter, prospective, randomized, controlled trial that included 207 patients demonstrated that patients treated orally for 1 year with *Lactobacillus casei* sp. *Shirota* together with transurethral epirubicin had a remarkably lower UBC recurrence rate at 3 years compared with the group treated with epirubicin only. However, overall survival did not differ between the groups [185]. In addition, a case-control study in 180 patients and 445 population-based controls revealed that regular (1–2 times per week) probiotic intake decreased UBC risk in the healthy population [178]. Taken together, these results strongly support the protective role of *Lactobacillus casei* sp. *Shirota* against bladder cancer. The bladder epithelium can work as a constant reservoir for viable but nonculturable uropathogenic bacterial strains, ultimately leading to bladder or kidney infection [156,186,187]. In these cases, *Escherichia coli*, *Klebsiella pneumoniae* and *Staphylococcus saprophyticus* strains are the most often isolated species. Still, many other bacteria can also be found [156,186,187,188], meaning that bladder commensal populations are polymicrobial and variable [160,182,183,189]. In bladder cancer, an initial study found a connection between urinary dysbiosis (specifically, an altered ratio among *Pseudomonas* and *Anaerococcus* versus *Streptococcus*) and urothelial carcinoma [171]. The urinary microbiota varies between men and women, and urinary dysbiosis might be associated with UBC. Indeed, the urinary microbiome might be different from the bacteria strictly related to the urothelium, and a clear association between mucosa-associated microbiota and the incidence and outcome of UBC is lacking.

## 3. Impact of the Microbiome on Gastroenteric and Genitourinary Cancers Treatment

Given the significant role that the microbiome appears to play in cancer, modulation of the microbiota could influence the course of the disease (Figure 1). Dysbiosis has so far been observed to play an important role in the onset of tumors. In particular, it was discussed how the presence or absence of some bacterial species can have a pro-cancerous or protective role. In response to these studies, it is easy to understand how a first therapeutic approach can be represented by pre, pro and postbiotics. Probiotics are live bacteria that can be administered orally [190] capable of improving the functionality of the intestinal barrier; prebiotics cannot be digested by the human body and are fermented by specific types of bacteria [191]; finally, postbiotics are bacterial products or metabolites that have beneficial activity within the human host [192]. A 2018 study showed how the alteration of the intestinal microbiome following an antibiotic treatment influences therapeutic responses, acting on antitumor immunity and the efficacy of immunotherapy [193]. The microbiota can act on the functionality of the therapy by modifying the pharmacokinetics of the drug used, thus altering its absorption, distribution, or metabolism [194]. Indeed, the structural and metabolic characteristics of bacteria recognize these potential targets to improve the efficacy of cancer therapy [195].

Changes in the gut microbiota are closely related to the side effects of chemotherapy and radiotherapy.

Chemotherapy with 5-fluorouracil reduces in the gut the abundance of pathogens such as *Enterobacteriaceae*. So, in a wild-type mouse model, supplementation with *Lactobacillus lactis* was engineered to secrete the protein associated with pancreatitis, an antimicrobial peptide involved in intestinal homeostasis, which might relieve the severity of mucositis.

Taken together, these data suggest that specific species of the gut microbiota induce antitumor responses and that associations between gut microbiota diversity and immunity indicate the exciting potential for the development of microbiome-based or additional therapeutic regimens for various malignancies, including cancers. Another very effective therapeutic strategy has been found in the transplantation of fecal microbiota (FMT) from a healthy subject to a sick subject. This method cauhuyses an alteration in the composition of the recipient’s intestinal microbiome. In reality, however, this strategy has not yet been approved by the FDA due to the various side effects presented (fever, diarrhea, vomiting, gastrointestinal bleeding or perforation). Furthermore, cancer patients have a depressed immune system, which is why donors should be screened for the presence of pathogens, including viral and fungal pathogens, which could cause infections after transplantation [196,197]. Finally, the composition of the microbiota is highly personalized, with many differences from individual to individual in the number of species, richness and localization within the gastrointestinal tract. Therefore, FMT treatment outcomes for cancer patients are likely to be both unpredictable and inconsistent [198]. However, microbiota transplantation appears to be a promising approach to further manipulating microbial composition and function to improve host antitumor immunity and to improve resistance and ineffectiveness in cancer patients with relatively short survival. These intestinal and intratumoral microflora will become future targets for overcoming oncogenesis and immunosuppression of the pancreas. In addition to FMT, a new therapeutic approach has been recognized in vaginal microbiota transplantation (VMT). In 2019, a pilot study was carried out on five women who underwent VMT. Of these five women, four participants witnessed a marked improvement in symptoms and the reconstruction of a microbiome after 21 months [199]. The study authors found no adverse effects after treatment, although the long-term consequences of this new therapeutic approach remain unknown. Enzymes and receptors can play an important role in the therapy of tumors in which the microbiota is involved [200]. An incorrect TLR signal can lead to a pathogenic immune response to a normal microbiota that can cause various diseases, including cancer [201]. This group of receptors could be a new therapeutic target for the treatment of tumors [135].

Β-glucuronidase enzymes are released by intestinal microbes and influence the metabolism of xenobiotics. Given the important role played by these enzymes and the high possibility of undergoing structural alterations, therapeutically engineered transgenic bacteria with modified proteins can be used to reduce the side effects of the drug [202]. While probiotics and FMT aim to promote anticancer responses by reconfiguring the gut microbiome, bacteria may also be effective cancer therapies outside of their role as commensal microorganisms. An area of interest lies in engineering bacteria for effective targeting of cancerous tissue and delivery of therapeutic loads, effectively transforming bacteria into anticancer factories. Bacteria can be readily transfected stably with vectors that code for many products, including RNAi [203,204], cytokines [205,206,207], toxins [208], antiangiogenic factors [209,210] and antibodies [211].

An advantage of using bacteria in this way is their rapid replication rate, which provides amplification of the transgene within the target microenvironment [212,213]. Anaerobic bacteria are particularly suitable for invading hypoxic tumor microenvironments. Bacteria such as *Shigella* and *Listeria* monocytogenes can enter the cytoplasm of mammalian host cells to deliver their engineered payloads, and some bacterial species also possess secretion systems, which can deliver therapeutic products into a target cell without the bacteria themselves entering the cell [214].

Engineered bacteria are also used to provide systemic anticancer immunity as they are capable of absorbing dangerous substances administered as a result of immunotherapies.

For example, Chowdhury et al. showed that tumor-infiltrating T cells could be activated using tumor-colonizing bacteria to deliver CD4nb and prevent metastasis, stimulating rapid tumor regression and leading to long-term survival. Tumor antigen-specific systemic immune responses that suppress the growth of untreated tumors are induced by local injection of CD4nb-expressing bacteria, demonstrating that engineered bacterial immunotherapy can have an abscopal effect [215].

However, this therapeutic strategy is not without risk. A subtle balance needs to be struck between the number of introduced bacteria needed to achieve a therapeutic effect and the number at which the bacteria overwhelm the host’s immune system, particularly in the context of common host immunosuppression in cancer patients.

## 4. Conclusions

Accumulated evidence indicates that microbiota can enhance the carcinogenesis process through alterations in the metabolism, by controlling epithelial proliferation and differentiation and by influencing the immune response towards pathogenic organisms. Dysbiosis due to an imbalance in the interactions between the host epithelia and the microbiota can initiate chronic inflammation, epithelial barrier dysfunction and overgrowth of harmful bacteria. The polymicrobial synergy creates an ideal microenvironment for tumor degeneration and the tumor-associated microbiome. MAMPs, PAMPs and DAMPs, interacting with each other with pro-inflammatory action, they might contribute to carcinogenesis, creating a “microbiota-cancer axis” recently defined oncobiome. Barrier defects and the presence of genotoxins and pro-tumor metabolites produced by bacteria are fundamental in the process of carcinogenesis. The gut microbiota influences the pathogenesis of gastroenteric cancers but also modulates the onset of genitourinary cancers. Genitourinary carcinogenesis is also influenced by the dysbiosis of two other microbiomes, the urinary one and the vaginal one. Finally, some oral bacteria have also been shown to confer augmented susceptibility to these neoplasms. Different anticancer approaches, such as the use of pre, pro and postbiotics, fecal or vaginal microbiota transplantation and engineering bacteria, act by counteracting dysbiosis.

## Data Availability

The study did not report any data.

## References

[1] Jandhyala S.M., Talukdar R., Subramanyam C., Vuyyuru H., Sasikala M., Nageshwar Reddy D. (2015). Role of the normal gut microbiota. World J. Gastroenterol..

[2] Thomas R.M., Jobin C. (2015). The Microbiome and Cancer: Is the ‘Oncobiome’ Mirage Real?. Trends Cancer.

[3] Schwabe R.F., Jobin C. (2013). The microbiome and cancer. Nat. Rev. Cancer.

[4] Oliva M., Mulet-Margalef N., Ochoa-De-Olza M., Napoli S., Mas J., Laquente B., Alemany L., Duell E.J., Nuciforo P., Moreno V. (2021). Tumor-Associated Microbiome: Where Do We Stand?. Int. J. Mol. Sci..

[5] Picardo S.L., Coburn B., Hansen A.R. (2019). The microbiome and cancer for clinicians. Crit. Rev. Oncol. Hematol..

[6] Pignatelli P., Iezzi L., Pennese M., Raimondi P., Cichella A., Bondi D., Grande R., Cotellese R., Di Bartolomeo N., Innocenti P. (2021). The Potential of Colonic Tumor Tissue. Cancers.

[7] Curia M.C., Fantini F., Lattanzio R., Tavano F., Di Mola F., Piantelli M., Battista P., Di Sebastiano P., Cama A. (2019). High methylation levels of PCDH10 predict poor prognosis in patients with pancreatic ductal adenocarcinoma. BMC Cancer.

[8] Hieken T.J., Chen J., Hoskin T.L., Walther-Antonio M., Johnson S., Ramaker S., Xiao J., Radisky D.C., Knutson K.L., Kalari K.R. (2016). The Microbiome of Aseptically Collected Human Breast Tissue in Benign and Malignant Disease. Sci. Rep..

[9] Pfirschke C., Garris C., Pittet M.J. (2015). Common TLR5 mutations control cancer progression. Cancer Cell.

[10] Pignatelli P., Fabietti G., Ricci A., Piattelli A., Curia M.C. (2020). How Periodontal Disease and Presence of Nitric Oxide Reducing Oral Bacteria Can Affect Blood Pressure. Int. J. Mol. Sci..

[11] Winter S.E., Winter M.G., Xavier M.N., Thiennimitr P., Poon V., Keestra A.M., Laughlin R.C., Gomez G., Wu J., Lawhon S.D. (2013). Host-derived nitrate boosts growth of E. coli in the inflamed gut. Science.

[12] Patwa L.G., Fan T.J., Tchaptchet S., Liu Y., Lussier Y.A., Sartor R.B., Hansen J.J. (2011). Chronic intestinal inflammation induces stress-response genes in commensal Escherichia coli. Gastroenterology.

[13] Andriamihaja M., Lan A., Beaumont M., Audebert M., Wong X., Yamada K., Yin Y., Tomé D., Carrasco-Pozo C., Gotteland M. (2015). The deleterious metabolic and genotoxic effects of the bacterial metabolite p-cresol on colonic epithelial cells. Free Radic. Biol. Med..

[14] Al Hinai E.A., Kullamethee P., Rowland I.R., Swann J., Walton G.E., Commane D.M. (2019). Modelling the role of microbial p-cresol in colorectal genotoxicity. Gut Microbes.

[15] Fukata M., Chen A., Vamadevan A.S., Cohen J., Breglio K., Krishnareddy S., Hsu D., Xu R., Harpaz N., Dannenberg A.J. (2007). Toll-like receptor-4 promotes the development of colitis-associated colorectal tumors. Gastroenterology.

[16] Ochi A., Graffeo C.S., Zambirinis C.P., Rehman A., Hackman M., Fallon N., Barilla R.M., Henning J.R., Jamal M., Rao R. (2012). Toll-like receptor 7 regulates pancreatic carcinogenesis in mice and humans. J. Clin. Investig..

[17] Moresco E.M., LaVine D., Beutler B. (2011). Toll-like receptors. Curr. Biol..

[18] Wells A.I., Coyne C.B. (2018). Type III Interferons in Antiviral Defenses at Barrier Surfaces. Trends Immunol..

[19] Hanahan D., Weinberg R.A. (2011). Hallmarks of cancer: The next generation. Cell.

[20] Kau A.L., Ahern P.P., Griffin N.W., Goodman A.L., Gordon J.I. (2011). Human nutrition, the gut microbiome and the immune system. Nature.

[21] Fraher M.H., O’Toole P.W., Quigley E.M. (2012). Techniques used to characterize the gut microbiota: A guide for the clinician. Nat. Rev. Gastroenterol. Hepatol..

[22] Colditz G.A., Sellers T.A., Trapido E. (2006). Epidemiology—Identifying the causes and preventability of cancer?. Nat. Rev. Cancer.

[23] Ragonnaud E., Biragyn A. (2021). Gut microbiota as the key controllers of “healthy” aging of elderly people. Immun. Ageing.

[24] Shiels P.G., Buchanan S., Selman C., Stenvinkel P. (2019). Allostatic load and ageing: Linking the microbiome and nutrition with age-related health. Biochem. Soc. Trans..

[25] Emanuele S., Celesia A., D’Anneo A., Lauricella M., Carlisi D., De Blasio A., Giuliano M. (2021). The Good and Bad of Nrf2: An Update in Cancer and New Perspectives in COVID-19. Int. J. Mol. Sci..

[26] Schmidlin C.J., Dodson M.B., Madhavan L., Zhang D.D. (2019). Redox regulation by NRF2 in aging and disease. Free. Radic. Biol. Med..

[27] Nukui A., Narimatsu T., Kambara T., Abe H., Sakamoto S., Yoshida K.I., Kamai T. (2018). Clinically significant association of elevated expression of nuclear factor E2-related factor 2 expression with higher glucose uptake and progression of upper urinary tract cancer. BMC Cancer.

[28] Hemmati M., Yousefi B., Bahar A., Eslami M. (2021). Importance of Heme Oxygenase-1 in Gastrointestinal Cancers: Functions, Inductions, Regulations, and Signaling. J. Gastrointest. Cancer.

[29] Li H., Christman L.M., Li R., Gu L. (2020). Synergic interactions between polyphenols and gut microbiota in mitigating inflammatory bowel diseases. Food Funct..

[30] Ramos-Molina B., Queipo-Ortuño M.I., Lambertos A., Tinahones F.J., Peñafiel R. (2019). Dietary and Gut Microbiota Polyamines in Obesity- and Age-Related Diseases. Front. Nutr..

[31] Franceschi C., Garagnani P., Parini P., Giuliani C., Santoro A. (2018). Inflammaging: A new immune-metabolic viewpoint for age-related diseases. Nat. Rev. Endocrinol..

[32] Martin A., Woolbright B.L., Umar S., Ingersoll M.A., Taylor J.A. (2022). Bladder cancer, inflammageing and microbiomes. Nat. Rev. Urol..

[33] Fox J.G., Wang T.C. (2007). Inflammation, atrophy, and gastric cancer. J. Clin. Investig..

[34] Lofgren J.L., Whary M.T., Ge Z., Muthupalani S., Taylor N.S., Mobley M., Potter A., Varro A., Eibach D., Suerbaum S. (2011). Lack of commensal flora in Helicobacter pylori-infected INS-GAS mice reduces gastritis and delays intraepithelial neoplasia. Gastroenterology.

[35] Bik E.M., Eckburg P.B., Gill S.R., Nelson K.E., Purdom E.A., Francois F., Perez-Perez G., Blaser M.J., Relman D.A. (2006). Molecular analysis of the bacterial microbiota in the human stomach. Proc. Natl. Acad. Sci. USA.

[36] Aviles-Jimenez F., Vazquez-Jimenez F., Medrano-Guzman R., Mantilla A., Torres J. (2014). Stomach microbiota composition varies between patients with non-atrophic gastritis and patients with intestinal type of gastric cancer. Sci. Rep..

[37] Stewart O.A., Wu F., Chen Y. (2020). The role of gastric microbiota in gastric cancer. Gut Microbes.

[38] Castaño-Rodríguez N., Goh K.L., Fock K.M., Mitchell H.M., Kaakoush N.O. (2017). Dysbiosis of the microbiome in gastric carcinogenesis. Sci. Rep..

[39] Chen Y., Peng Y., Yu J., Chen T., Wu Y., Shi L., Li Q., Wu J., Fu X. (2017). Invasive Fusobacterium nucleatum activates beta-catenin signaling in colorectal cancer via a TLR4/P-PAK1 cascade. Oncotarget.

[40] Liu X., Shao L., Ji F., Mei Y., Cheng Y., Liu F., Yan C., Li L., Ling Z. (2019). Alterations of gastric mucosal microbiota across different stomach microhabitats in a cohort of 276 patients with gastric cancer. EBioMedicine.

[41] Hu Y.L., Pang W., Huang Y., Zhang Y., Zhang C.J. (2018). The Gastric Microbiome Is Perturbed in Advanced Gastric Adenocarcinoma Identified Through Shotgun Metagenomics. Front. Cell. Infect. Microbiol..

[42] Liang W., Yang Y., Wang H., Yu X., Lu Y., Shen S., Teng L. (2019). Gut microbiota shifts in patients with gastric cancer in perioperative period. Medicine.

[43] Siegel R.L., Miller K.D., Jemal A. (2019). Cancer statistics, 2019. CA Cancer J. Clin..

[44] Maekawa T., Fukaya R., Takamatsu S., Itoyama S., Fukuoka T., Yamada M., Hata T., Nagaoka S., Kawamoto K., Eguchi H. (2018). Possible involvement of Enterococcus infection in the pathogenesis of chronic pancreatitis and cancer. Biochem. Biophys. Res. Commun..

[45] Ertz-Archambault N., Keim P., Von Hoff D. (2017). Microbiome and pancreatic cancer: A comprehensive topic review of literature. World J. Gastroenterol..

[46] McAllister F., Khan M.A.W., Helmink B., Wargo J.A. (2019). The Tumor Microbiome in Pancreatic Cancer: Bacteria and Beyond. Cancer Cell.

[47] Lloyd-Price J., Mahurkar A., Rahnavard G., Crabtree J., Orvis J., Hall A.B., Brady A., Creasy H.H., McCracken C., Giglio M.G. (2017). Strains, functions and dynamics in the expanded Human Microbiome Project. Nature.

[48] Pushalkar S., Hundeyin M., Daley D., Zambirinis C.P., Kurz E., Mishra A., Mohan N., Aykut B., Usyk M., Torres L.E. (2018). The Pancreatic Cancer Microbiome Promotes Oncogenesis by Induction of Innate and Adaptive Immune Suppression. Cancer Discov..

[49] Sethi V., Kurtom S., Tarique M., Lavania S., Malchiodi Z., Hellmund L., Zhang L., Sharma U., Giri B., Garg B. (2018). Gut Microbiota Promotes Tumor Growth in Mice by Modulating Immune Response. Gastroenterology.

[50] Ren Z., Jiang J., Xie H., Li A., Lu H., Xu S., Zhou L., Zhang H., Cui G., Chen X. (2017). Gut microbial profile analysis by MiSeq sequencing of pancreatic carcinoma patients in China. Oncotarget.

[51] Lu H., Ren Z., Li A., Li J., Xu S., Zhang H., Jiang J., Yang J., Luo Q., Zhou K. (2019). Tongue coating microbiome data distinguish patients with pancreatic head cancer from healthy controls. J. Oral Microbiol..

[52] Sun H., Zhao X., Zhou Y., Wang J., Ma R., Ren X., Wang H., Zou L. (2020). Characterization of Oral Microbiome and Exploration of Potential Biomarkers in Patients with Pancreatic Cancer. BioMed Res. Int..

[53] Michaud D.S., Izard J., Wilhelm-Benartzi C.S., You D.H., Grote V.A., Tjønneland A., Dahm C.C., Overvad K., Jenab M., Fedirko V. (2013). Plasma antibodies to oral bacteria and risk of pancreatic cancer in a large European prospective cohort study. Gut.

[54] Riquelme E., Zhang Y., Zhang L., Montiel M., Zoltan M., Dong W., Quesada P., Sahin I., Chandra V., San Lucas A. (2019). Tumor Microbiome Diversity and Composition Influence Pancreatic Cancer Outcomes. Cell.

[55] Cani P.D. (2017). Gut microbiota—At the intersection of everything?. Nat. Rev. Gastroenterol. Hepatol..

[56] Sears C.L., Garrett W.S. (2014). Microbes, microbiota, and colon cancer. Cell Host Microbe.

[57] Flynn K.J., Baxter N.T., Schloss P.D. (2016). Metabolic and Community Synergy of Oral Bacteria in Colorectal Cancer. mSphere.

[58] Sekirov I., Russell S.L., Antunes L.C., Finlay B.B. (2010). Gut microbiota in health and disease. Physiol. Rev..

[59] Gao X., Jia R., Xie L., Kuang L., Feng L., Wan C. (2015). Obesity in school-aged children and its correlation with gut E.coli and Bifidobacteria: A case-control study. BMC Pediatr..

[60] Marchesi J.R. (2011). Human distal gut microbiome. Environ. Microbiol..

[61] Zhang Y.K., Zhang Q., Wang Y.L., Zhang W.Y., Hu H.Q., Wu H.Y., Sheng X.Z., Luo K.J., Zhang H., Wang M. (2021). A Comparison Study of Age and Colorectal Cancer-Related Gut Bacteria. Front. Cell. Infect. Microbiol..

[62] Baxter N.T., Zackular J.P., Chen G.Y., Schloss P.D. (2014). Structure of the gut microbiome following colonization with human feces determines colonic tumor burden. Microbiome.

[63] Chung L., Orberg E.T., Geis A.L., Chan J.L., Fu K., DeStefano Shields C.E., Dejea C.M., Fathi P., Chen J., Finard B.B. (2018). Bacteroides fragilis Toxin Coordinates a Pro-carcinogenic Inflammatory Cascade via Targeting of Colonic Epithelial Cells. Cell Host Microbe.

[64] Liu X., Cheng Y., Shao L., Ling Z. (2020). Alterations of the Predominant Fecal Microbiota and Disruption of the Gut Mucosal Barrier in Patients with Early-Stage Colorectal Cancer. BioMed Res. Int..

[65] Chervy M., Barnich N., Denizot J. (2020). Adherent-Invasive. Int. J. Mol. Sci..

[66] Capaldo C.T., Powell D.N., Kalman D. (2017). Layered defense: How mucus and tight junctions seal the intestinal barrier. J. Mol. Med..

[67] Gil-Cardoso K., Comitato R., Ginés I., Ardévol A., Pinent M., Virgili F., Terra X., Blay M. (2019). Protective Effect of Proanthocyanidins in a Rat Model of Mild Intestinal Inflammation and Impaired Intestinal Permeability Induced by LPS. Mol. Nutr. Food Res..

[68] Rubinstein M.R., Wang X., Liu W., Hao Y., Cai G., Han Y.W. (2013). Fusobacterium nucleatum promotes colorectal carcinogenesis by modulating E-cadherin/β-catenin signaling via its FadA adhesin. Cell Host Microbe.

[69] Casasanta M.A., Yoo C.C., Udayasuryan B., Sanders B.E., Umaña A., Zhang Y., Peng H., Duncan A.J., Wang Y., Li L. (2020). Host-cell binding and invasion induces IL-8 and CXCL1 secretion that drives colorectal cancer cell migration. Sci. Signal..

[70] Rubinstein M.R., Baik J.E., Lagana S.M., Han R.P., Raab W.J., Sahoo D., Dalerba P., Wang T.C., Han Y.W. (2019). promotes colorectal cancer by inducing Wnt/β-catenin modulator Annexin A1. EMBO Rep..

[71] Vancamelbeke M., Vermeire S. (2017). The intestinal barrier: A fundamental role in health and disease. Expert Rev. Gastroenterol. Hepatol..

[72] Dalal N., Jalandra R., Bayal N., Yadav A.K., Harshulika, Sharma M., Makharia G.K., Kumar P., Singh R., Solanki P.R. (2021). Gut microbiota-derived metabolites in CRC progression and causation. J. Cancer Res. Clin. Oncol..

[73] Osadchiy V., Martin C.R., Mayer E.A. (2019). The Gut-Brain Axis and the Microbiome: Mechanisms and Clinical Implications. Clin. Gastroenterol. Hepatol..

[74] Alpert O., Begun L., Issac T., Solhkhah R. (2021). The brain-gut axis in gastrointestinal cancers. J. Gastrointest. Oncol..

[75] Furness J.B., Rivera L.R., Cho H.J., Bravo D.M., Callaghan B. (2013). The gut as a sensory organ. Nat. Rev. Gastroenterol. Hepatol..

[76] Tolhurst G., Heffron H., Lam Y.S., Parker H.E., Habib A.M., Diakogiannaki E., Cameron J., Grosse J., Reimann F., Gribble F.M. (2012). Short-chain fatty acids stimulate glucagon-like peptide-1 secretion via the G-protein-coupled receptor FFAR2. Diabetes.

[77] Yano J.M., Yu K., Donaldson G.P., Shastri G.G., Ann P., Ma L., Nagler C.R., Ismagilov R.F., Mazmanian S.K., Hsiao E.Y. (2015). Indigenous bacteria from the gut microbiota regulate host serotonin biosynthesis. Cell.

[78] Haghikia A., Jörg S., Duscha A., Berg J., Manzel A., Waschbisch A., Hammer A., Lee D.H., May C., Wilck N. (2015). Dietary Fatty Acids Directly Impact Central Nervous System Autoimmunity via the Small Intestine. Immunity.

[79] Kim D.Y., Camilleri M. (2000). Serotonin: A mediator of the brain-gut connection. Am. J. Gastroenterol..

[80] Ruddick J.P., Evans A.K., Nutt D.J., Lightman S.L., Rook G.A., Lowry C.A. (2006). Tryptophan metabolism in the central nervous system: Medical implications. Expert Rev. Mol. Med..

[81] Amit M., Na’ara S., Gil Z. (2016). Mechanisms of cancer dissemination along nerves. Nat. Rev. Cancer.

[82] Wieczorska K., Stolarek M., Stec R. (2020). The Role of the Gut Microbiome in Colorectal Cancer: Where Are We? Where Are We Going?. Clin. Colorectal Cancer.

[83] Mayer E.A., Nance K., Chen S. (2022). The Gut-Brain Axis. Annu. Rev. Med..

[84] Raskov H., Burcharth J., Pommergaard H.C., Rosenberg J. (2016). Irritable bowel syndrome, the microbiota and the gut-brain axis. Gut Microbes.

[85] Baker J.M., Al-Nakkash L., Herbst-Kralovetz M.M. (2017). Estrogen-gut microbiome axis: Physiological and clinical implications. Maturitas.

[86] Thomas-White K., Forster S.C., Kumar N., Van Kuiken M., Putonti C., Stares M.D., Hilt E.E., Price T.K., Wolfe A.J., Lawley T.D. (2018). Culturing of female bladder bacteria reveals an interconnected urogenital microbiota. Nat. Commun..

[87] Plottel C.S., Blaser M.J. (2011). Microbiome and malignancy. Cell Host Microbe.

[88] Ravel J., Gajer P., Abdo Z., Schneider G.M., Koenig S.S., McCulle S.L., Karlebach S., Gorle R., Russell J., Tacket C.O. (2011). Vaginal microbiome of reproductive-age women. Proc. Natl. Acad. Sci. USA.

[89] Martin D.H., Marrazzo J.M. (2016). The Vaginal Microbiome: Current Understanding and Future Directions. J. Infect. Dis..

[90] Beamer M.A., Austin M.N., Avolia H.A., Meyn L.A., Bunge K.E., Hillier S.L. (2017). Bacterial species colonizing the vagina of healthy women are not associated with race. Anaerobe.

[91] Mirmonsef P., Hotton A.L., Gilbert D., Gioia C.J., Maric D., Hope T.J., Landay A.L., Spear G.T. (2016). Glycogen Levels in Undiluted Genital Fluid and Their Relationship to Vaginal pH, Estrogen, and Progesterone. PLoS ONE.

[92] Łaniewski P., Barnes D., Goulder A., Cui H., Roe D.J., Chase D.M., Herbst-Kralovetz M.M. (2018). Linking cervicovaginal immune signatures, HPV and microbiota composition in cervical carcinogenesis in non-Hispanic and Hispanic women. Sci. Rep..

[93] Hickey R.J., Zhou X., Pierson J.D., Ravel J., Forney L.J. (2012). Understanding vaginal microbiome complexity from an ecological perspective. Transl. Res..

[94] Maldonado-Barragán A., Caballero-Guerrero B., Martín V., Ruiz-Barba J.L., Rodríguez J.M. (2016). Purification and genetic characterization of gassericin E, a novel co-culture inducible bacteriocin from Lactobacillus gasseri EV1461 isolated from the vagina of a healthy woman. BMC Microbiol..

[95] Graver M.A., Wade J.J. (2011). The role of acidification in the inhibition of Neisseria gonorrhoeae by vaginal lactobacilli during anaerobic growth. Ann. Clin. Microbiol. Antimicrob..

[96] Gong Z., Luna Y., Yu P., Fan H. (2014). Lactobacilli inactivate Chlamydia trachomatis through lactic acid but not H2O2. PLoS ONE.

[97] Conti C., Malacrino C., Mastromarino P. (2009). Inhibition of herpes simplex virus type 2 by vaginal lactobacilli. J. Physiol. Pharmacol..

[98] Tyssen D., Wang Y.Y., Hayward J.A., Agius P.A., DeLong K., Aldunate M., Ravel J., Moench T.R., Cone R.A., Tachedjian G. (2018). Anti-HIV-1 Activity of Lactic Acid in Human Cervicovaginal Fluid. mSphere.

[99] Cadieux P.A., Burton J., Devillard E., Reid G. (2009). Lactobacillus by-products inhibit the growth and virulence of uropathogenic Escherichia coli. J. Physiol. Pharmacol..

[100] Han Y.W., Redline R.W., Li M., Yin L., Hill G.B., McCormick T.S. (2004). Fusobacterium nucleatum induces premature and term stillbirths in pregnant mice: Implication of oral bacteria in preterm birth. Infect. Immun..

[101] Lindheim L., Bashir M., Münzker J., Trummer C., Zachhuber V., Leber B., Horvath A., Pieber T.R., Gorkiewicz G., Stadlbauer V. (2017). Alterations in Gut Microbiome Composition and Barrier Function Are Associated with Reproductive and Metabolic Defects in Women with Polycystic Ovary Syndrome (PCOS): A Pilot Study. PLoS ONE.

[102] Lewis F.M.T., Bernstein K.T., Aral S.O. (2017). Vaginal Microbiome and Its Relationship to Behavior, Sexual Health, and Sexually Transmitted Diseases. Obstet. Gynecol..

[103] Serrano M.G., Parikh H.I., Brooks J.P., Edwards D.J., Arodz T.J., Edupuganti L., Huang B., Girerd P.H., Bokhari Y.A., Bradley S.P. (2019). Racioethnic diversity in the dynamics of the vaginal microbiome during pregnancy. Nat. Med..

[104] Muhleisen A.L., Herbst-Kralovetz M.M. (2016). Menopause and the vaginal microbiome. Maturitas.

[105] Romero R., Hassan S.S., Gajer P., Tarca A.L., Fadrosh D.W., Bieda J., Chaemsaithong P., Miranda J., Chaiworapongsa T., Ravel J. (2014). The vaginal microbiota of pregnant women who subsequently have spontaneous preterm labor and delivery and those with a normal delivery at term. Microbiome.

[106] Jespers V., Kyongo J., Joseph S., Hardy L., Cools P., Crucitti T., Mwaura M., Ndayisaba G., Delany-Moretlwe S., Buyze J. (2017). A longitudinal analysis of the vaginal microbiota and vaginal immune mediators in women from sub-Saharan Africa. Sci. Rep..

[107] Gajer P., Brotman R.M., Bai G., Sakamoto J., Schütte U.M., Zhong X., Koenig S.S., Fu L., Ma Z.S., Zhou X. (2012). Temporal dynamics of the human vaginal microbiota. Sci. Transl. Med..

[108] Witkin S.S., Mendes-Soares H., Linhares I.M., Jayaram A., Ledger W.J., Forney L.J. (2013). Influence of vaginal bacteria and D- and L-lactic acid isomers on vaginal extracellular matrix metalloproteinase inducer: Implications for protection against upper genital tract infections. mBio.

[109] Borgdorff H., van der Veer C., van Houdt R., Alberts C.J., de Vries H.J., Bruisten S.M., Snijder M.B., Prins M., Geerlings S.E., Schim van der Loeff M.F. (2017). The association between ethnicity and vaginal microbiota composition in Amsterdam, the Netherlands. PLoS ONE.

[110] Chen C., Song X., Wei W., Zhong H., Dai J., Lan Z., Li F., Yu X., Feng Q., Wang Z. (2017). The microbiota continuum along the female reproductive tract and its relation to uterine-related diseases. Nat. Commun..

[111] Ingerslev K., Hogdall E., Schnack T.H., Skovrider-Ruminski W., Hogdall C., Blaakaer J. (2017). The potential role of infectious agents and pelvic inflammatory disease in ovarian carcinogenesis. Infect. Agents Cancer.

[112] Farhana L., Banerjee H.N., Verma M., Majumdar A.P.N. (2018). Role of Microbiome in Carcinogenesis Process and Epigenetic Regulation of Colorectal Cancer. Methods Mol. Biol..

[113] Helmink B.A., Khan M.A.W., Hermann A., Gopalakrishnan V., Wargo J.A. (2019). The microbiome, cancer, and cancer therapy. Nat. Med..

[114] Scott A.J., Alexander J.L., Merrifield C.A., Cunningham D., Jobin C., Brown R., Alverdy J., O’Keefe S.J., Gaskins H.R., Teare J. (2019). International Cancer Microbiome Consortium consensus statement on the role of the human microbiome in carcinogenesis. Gut.

[115] Bhatt A.P., Redinbo M.R., Bultman S.J. (2017). The role of the microbiome in cancer development and therapy. CA Cancer J. Clin..

[116] Barrila J., Radtke A.L., Crabbé A., Sarker S.F., Herbst-Kralovetz M.M., Ott C.M., Nickerson C.A. (2010). Organotypic 3D cell culture models: Using the rotating wall vessel to study host-pathogen interactions. Nat. Rev. Microbiol..

[117] Audirac-Chalifour A., Torres-Poveda K., Bahena-Román M., Téllez-Sosa J., Martínez-Barnetche J., Cortina-Ceballos B., López-Estrada G., Delgado-Romero K., Burguete-García A.I., Cantú D. (2016). Cervical Microbiome and Cytokine Profile at Various Stages of Cervical Cancer: A Pilot Study. PLoS ONE.

[118] Kovachev S.M. (2020). Cervical cancer and vaginal microbiota changes. Arch. Microbiol..

[119] Amabebe E., Anumba D.O.C. (2018). The Vaginal Microenvironment: The Physiologic Role of. Front. Med..

[120] Spear G.T., French A.L., Gilbert D., Zariffard M.R., Mirmonsef P., Sullivan T.H., Spear W.W., Landay A., Micci S., Lee B.H. (2014). Human α-amylase present in lower-genital-tract mucosal fluid processes glycogen to support vaginal colonization by Lactobacillus. J. Infect. Dis..

[121] Dossus L., Rinaldi S., Becker S., Lukanova A., Tjonneland A., Olsen A., Stegger J., Overvad K., Chabbert-Buffet N., Jimenez-Corona A. (2010). Obesity, inflammatory markers, and endometrial cancer risk: A prospective case-control study. Endocr.-Relat. Cancer.

[122] Tilg H., Moschen A.R., Kaser A. (2009). Obesity and the microbiota. Gastroenterology.

[123] Chase D., Goulder A., Zenhausern F., Monk B., Herbst-Kralovetz M. (2015). The vaginal and gastrointestinal microbiomes in gynecologic cancers: A review of applications in etiology, symptoms and treatment. Gynecol. Oncol..

[124] Marconi C., Cruciani F., Vitali B., Donders G.G. (2012). Correlation of Atopobium vaginae Amount With Bacterial Vaginosis Markers. J. Low. Genit. Tract Dis..

[125] Walther-António M.R., Chen J., Multinu F., Hokenstad A., Distad T.J., Cheek E.H., Keeney G.L., Creedon D.J., Nelson H., Mariani A. (2016). Potential contribution of the uterine microbiome in the development of endometrial cancer. Genome Med..

[126] Shanmughapriya S., Senthilkumar G., Vinodhini K., Das B.C., Vasanthi N., Natarajaseenivasan K. (2012). Viral and bacterial aetiologies of epithelial ovarian cancer. Eur. J. Clin. Microbiol. Infect. Dis..

[127] Zhou B., Sun C., Huang J., Xia M., Guo E., Li N., Lu H., Shan W., Wu Y., Li Y. (2019). The biodiversity Composition of Microbiome in Ovarian Carcinoma Patients. Sci. Rep..

[128] Reid B.M., Permuth J.B., Sellers T.A. (2017). Epidemiology of ovarian cancer: A review. Cancer Biol. Med..

[129] Tong J., Zhang X., Fan Y., Chen L., Ma X., Yu H., Li J., Guan X., Zhao P., Yang J. (2020). Changes of Intestinal Microbiota in Ovarian Cancer Patients Treated with Surgery and Chemotherapy. Cancer Manag. Res..

[130] Banerjee S., Tian T., Wei Z., Shih N., Feldman M.D., Alwine J.C., Coukos G., Robertson E.S. (2017). The ovarian cancer oncobiome. Oncotarget.

[131] Chan P.J., Seraj I.M., Kalugdan T.H., King A. (1996). Prevalence of mycoplasma conserved DNA in malignant ovarian cancer detected using sensitive PCR-ELISA. Gynecol. Oncol..

[132] Zeng W., Shen J., Bo T., Peng L., Xu H., Nasser M.I., Zhuang Q., Zhao M. (2019). Cutting Edge: Probiotics and Fecal Microbiota Transplantation in Immunomodulation. J. Immunol. Res..

[133] Trabert B., Poole E.M., White E., Visvanathan K., Adami H.O., Anderson G.L., Brasky T.M., Brinton L.A., Fortner R.T., Gaudet M. (2019). Analgesic Use and Ovarian Cancer Risk: An Analysis in the Ovarian Cancer Cohort Consortium. J. Natl. Cancer Inst..

[134] Di Giovanni S.E., Cunha T.M., Duarte A.L., Alves I. (2016). Endometrial Tuberculosis Simulating an Ovarian Cancer: A case report. Acta Med. Port..

[135] Dong Q., Nelson D.E., Toh E., Diao L., Gao X., Fortenberry J.D., Van der Pol B. (2011). The microbial communities in male first catch urine are highly similar to those in paired urethral swab specimens. PLoS ONE.

[136] Nelson D.E., Dong Q., Van der Pol B., Toh E., Fan B., Katz B.P., Mi D., Rong R., Weinstock G.M., Sodergren E. (2012). Bacterial communities of the coronal sulcus and distal urethra of adolescent males. PLoS ONE.

[137] Nelson D.E., Van Der Pol B., Dong Q., Revanna K.V., Fan B., Easwaran S., Sodergren E., Weinstock G.M., Diao L., Fortenberry J.D. (2010). Characteristic male urine microbiomes associate with asymptomatic sexually transmitted infection. PLoS ONE.

[138] Ferlay J., Colombet M., Soerjomataram I., Mathers C., Parkin D.M., Piñeros M., Znaor A., Bray F. (2019). Estimating the global cancer incidence and mortality in 2018: GLOBOCAN sources and methods. Int. J. Cancer.

[139] De Marzo A.M., Nakai Y., Nelson W.G. (2007). Inflammation, atrophy, and prostate carcinogenesis. Urol. Oncol..

[140] Sfanos K.S., De Marzo A.M. (2012). Prostate cancer and inflammation: The evidence. Histopathology.

[141] Cavarretta I., Ferrarese R., Cazzaniga W., Saita D., Lucianò R., Ceresola E.R., Locatelli I., Visconti L., Lavorgna G., Briganti A. (2017). The Microbiome of the Prostate Tumor Microenvironment. Eur. Urol..

[142] Feng Y., Ramnarine V.R., Bell R., Volik S., Davicioni E., Hayes V.M., Ren S., Collins C.C. (2019). Metagenomic and metatranscriptomic analysis of human prostate microbiota from patients with prostate cancer. BMC Genom..

[143] Shoskes D.A., Altemus J., Polackwich A.S., Tucky B., Wang H., Eng C. (2016). The Urinary Microbiome Differs Significantly Between Patients With Chronic Prostatitis/Chronic Pelvic Pain Syndrome and Controls as Well as Between Patients With Different Clinical Phenotypes. Urology.

[144] Bajic P., Dornbier R.A., Doshi C.P., Wolfe A.J., Farooq A.V., Bresler L. (2019). Implications of the Genitourinary Microbiota in Prostatic Disease. Curr. Urol. Rep..

[145] Markowski M.C., Boorjian S.A., Burton J.P., Hahn N.M., Ingersoll M.A., Maleki Vareki S., Pal S.K., Sfanos K.S. (2019). The Microbiome and Genitourinary Cancer: A Collaborative Review. Eur. Urol..

[146] Shrestha E., White J.R., Yu S.H., Kulac I., Ertunc O., De Marzo A.M., Yegnasubramanian S., Mangold L.A., Partin A.W., Sfanos K.S. (2018). Profiling the Urinary Microbiome in Men with Positive versus Negative Biopsies for Prostate Cancer. J. Urol..

[147] Yu Y., Sikorski P., Bowman-Gholston C., Cacciabeve N., Nelson K.E., Pieper R. (2015). Diagnosing inflammation and infection in the urinary system via proteomics. J. Transl. Med..

[148] Nickel J.C., Stephens A., Landis J.R., Mullins C., van Bokhoven A., Lucia M.S., Ehrlich G.D., Network M.R. (2016). Assessment of the Lower Urinary Tract Microbiota during Symptom Flare in Women with Urologic Chronic Pelvic Pain Syndrome: A MAPP Network Study. J. Urol..

[149] Alanee S., El-Zawahry A., Dynda D., Dabaja A., McVary K., Karr M., Braundmeier-Fleming A. (2019). A prospective study to examine the association of the urinary and fecal microbiota with prostate cancer diagnosis after transrectal biopsy of the prostate using 16sRNA gene analysis. Prostate.

[150] Karstens L., Asquith M., Caruso V., Rosenbaum J.T., Fair D.A., Braun J., Gregory W.T., Nardos R., McWeeney S.K. (2018). Community profiling of the urinary microbiota: Considerations for low-biomass samples. Nat. Rev. Urol..

[151] Golombos D.M., Ayangbesan A., O’Malley P., Lewicki P., Barlow L., Barbieri C.E., Chan C., DuLong C., Abu-Ali G., Huttenhower C. (2018). The Role of Gut Microbiome in the Pathogenesis of Prostate Cancer: A Prospective, Pilot Study. Urology.

[152] Aune D., Navarro Rosenblatt D.A., Chan D.S., Vieira A.R., Vieira R., Greenwood D.C., Vatten L.J., Norat T. (2015). Dairy products, calcium, and prostate cancer risk: A systematic review and meta-analysis of cohort studies. Am. J. Clin. Nutr..

[153] Reed J.P., Devkota S., Figlin R.A. (2019). Gut microbiome, antibiotic use, and immunotherapy responsiveness in cancer. Ann. Transl. Med..

[154] Sfanos K.S., Yegnasubramanian S., Nelson W.G., De Marzo A.M. (2018). The inflammatory microenvironment and microbiome in prostate cancer development. Nat. Rev. Urol..

[155] Aragón I.M., Herrera-Imbroda B., Queipo-Ortuño M.I., Castillo E., Del Moral J.S., Gómez-Millán J., Yucel G., Lara M.F. (2018). The Urinary Tract Microbiome in Health and Disease. Eur. Urol. Focus.

[156] Fouts D.E., Pieper R., Szpakowski S., Pohl H., Knoblach S., Suh M.J., Huang S.T., Ljungberg I., Sprague B.M., Lucas S.K. (2012). Integrated next-generation sequencing of 16S rDNA and metaproteomics differentiate the healthy urine microbiome from asymptomatic bacteriuria in neuropathic bladder associated with spinal cord injury. J. Transl. Med..

[157] Zozaya M., Ferris M.J., Siren J.D., Lillis R., Myers L., Nsuami M.J., Eren A.M., Brown J., Taylor C.M., Martin D.H. (2016). Bacterial communities in penile skin, male urethra, and vaginas of heterosexual couples with and without bacterial vaginosis. Microbiome.

[158] Altmäe S., Franasiak J.M., Mändar R. (2019). The seminal microbiome in health and disease. Nat. Rev. Urol..

[159] Lewis D.A., Brown R., Williams J., White P., Jacobson S.K., Marchesi J.R., Drake M.J. (2013). The human urinary microbiome; bacterial DNA in voided urine of asymptomatic adults. Front. Cell. Infect. Microbiol..

[160] Siddiqui H., Nederbragt A.J., Lagesen K., Jeansson S.L., Jakobsen K.S. (2011). Assessing diversity of the female urine microbiota by high throughput sequencing of 16S rDNA amplicons. BMC Microbiol..

[161] Arumugam M., Raes J., Pelletier E., Le Paslier D., Yamada T., Mende D.R., Fernandes G.R., Tap J., Bruls T., Batto J.M. (2011). Enterotypes of the human gut microbiome. Nature.

[162] Jalanka-Tuovinen J., Salonen A., Nikkilä J., Immonen O., Kekkonen R., Lahti L., Palva A., de Vos W.M. (2011). Intestinal microbiota in healthy adults: Temporal analysis reveals individual and common core and relation to intestinal symptoms. PLoS ONE.

[163] Bray F., Ferlay J., Soerjomataram I., Siegel R.L., Torre L.A., Jemal A. (2018). Global cancer statistics 2018: GLOBOCAN estimates of incidence and mortality worldwide for 36 cancers in 185 countries. CA Cancer J. Clin..

[164] Cosma C.L., Sherman D.R., Ramakrishnan L. (2003). The secret lives of the pathogenic mycobacteria. Annu. Rev. Microbiol..

[165] Curtiss N., Balachandran A., Krska L., Peppiatt-Wildman C., Wildman S., Duckett J. (2018). Age, menopausal status and the bladder microbiome. Eur. J. Obstet. Gynecol. Reprod. Biol..

[166] Wolfe A.J., Toh E., Shibata N., Rong R., Kenton K., Fitzgerald M., Mueller E.R., Schreckenberger P., Dong Q., Nelson D.E. (2012). Evidence of uncultivated bacteria in the adult female bladder. J. Clin. Microbiol..

[167] Andolfi C., Gundeti M.S. (2020). Live-case demonstrations in pediatric urology: Ethics, patient safety, and clinical outcomes from an 8-year institutional experience. Investig. Clin. Urol..

[168] Akram A.M., Iqbal Z., Akhtar T., Khalid A.M., Sabar M.F., Qazi M.H., Aziz Z., Sajid N., Aleem A., Rasool M. (2017). Presence of novel compound BCR-ABL mutations in late chronic and advanced phase imatinib sensitive CML patients indicates their possible role in CML progression. Cancer Biol. Ther..

[169] Knowles M.A., Hurst C.D. (2015). Molecular biology of bladder cancer: New insights into pathogenesis and clinical diversity. Nat. Rev. Cancer.

[170] Honeycutt J., Hammam O., Fu C.L., Hsieh M.H. (2014). Controversies and challenges in research on urogenital schistosomiasis-associated bladder cancer. Trends Parasitol..

[171] Xu W., Yang L., Lee P., Huang W.C., Nossa C., Ma Y., Deng F.M., Zhou M., Melamed J., Pei Z. (2014). Mini-review: Perspective of the microbiome in the pathogenesis of urothelial carcinoma. Am. J. Clin. Exp. Urol..

[172] Bučević Popović V., Šitum M., Chow C.T., Chan L.S., Roje B., Terzić J. (2018). The urinary microbiome associated with bladder cancer. Sci. Rep..

[173] Wu C.E., Lin Y.C., Hong J.H., Chuang C.K., Pang S.T., Liaw C.C. (2013). Prognostic value of complete response in patients with muscle-invasive bladder cancer undergoing concurrent chemoradiotherapy. Anticancer. Res..

[174] McConnell M.J., Actis L., Pachón J. (2013). Acinetobacter baumannii: Human infections, factors contributing to pathogenesis and animal models. FEMS Microbiol. Rev..

[175] Murphy E.C., Frick I.M. (2013). Gram-positive anaerobic cocci--commensals and opportunistic pathogens. FEMS Microbiol. Rev..

[176] Alfano M., Canducci F., Nebuloni M., Clementi M., Montorsi F., Salonia A. (2016). The interplay of extracellular matrix and microbiome in urothelial bladder cancer. Nat. Rev. Urol..

[177] Seow S.W., Rahmat J.N., Mohamed A.A., Mahendran R., Lee Y.K., Bay B.H. (2002). Lactobacillus species is more cytotoxic to human bladder cancer cells than Mycobacterium Bovis (bacillus Calmette-Guerin). J. Urol..

[178] Ohashi Y., Nakai S., Tsukamoto T., Masumori N., Akaza H., Miyanaga N., Kitamura T., Kawabe K., Kotake T., Kuroda M. (2002). Habitual intake of lactic acid bacteria and risk reduction of bladder cancer. Urol. Int..

[179] Babjuk M., Böhle A., Burger M., Capoun O., Cohen D., Compérat E.M., Hernández V., Kaasinen E., Palou J., Rouprêt M. (2017). EAU Guidelines on Non-Muscle-invasive Urothelial Carcinoma of the Bladder: Update 2016. Eur. Urol..

[180] Witjes J.A. (2016). Bladder cancer in 2015: Improving indication, technique and outcome of radical cystectomy. Nat. Rev. Urol..

[181] Shida K., Nomoto K. (2013). Probiotics as efficient immunopotentiators: Translational role in cancer prevention. Indian J. Med. Res..

[182] Takagi A., Matsuzaki T., Sato M., Nomoto K., Morotomi M., Yokokura T. (2001). Enhancement of natural killer cytotoxicity delayed murine carcinogenesis by a probiotic microorganism. Carcinogenesis.

[183] Matsumoto S., Shimizu N., Hanai T., Uemura H., Levin R. (2009). Bladder outlet obstruction accelerates bladder carcinogenesis. BJU Int..

[184] Aso Y., Akaza H., Kotake T., Tsukamoto T., Imai K., Naito S. (1995). Preventive effect of a Lactobacillus casei preparation on the recurrence of superficial bladder cancer in a double-blind trial. The BLP Study Group. Eur. Urol..

[185] Naito S., Tsukamoto T., Koga H., Harabayashi T., Sumiyoshi Y., Hoshi S., Akaza H. (2008). Docetaxel plus prednisolone for the treatment of metastatic hormone-refractory prostate cancer: A multicenter Phase II trial in Japan. Jpn. J. Clin. Oncol..

[186] Anderson G.G., Dodson K.W., Hooton T.M., Hultgren S.J. (2004). Intracellular bacterial communities of uropathogenic Escherichia coli in urinary tract pathogenesis. Trends Microbiol..

[187] Schilling J.D., Hultgren S.J., Lorenz R.G. (2002). Recent advances in the molecular basis of pathogen recognition and host responses in the urinary tract. Int. Rev. Immunol..

[188] Ronald A. (2003). The etiology of urinary tract infection: Traditional and emerging pathogens. Dis. Mon..

[189] Guinane C.M., Tadrous A., Fouhy F., Ryan C.A., Dempsey E.M., Murphy B., Andrews E., Cotter P.D., Stanton C., Ross R.P. (2013). Microbial composition of human appendices from patients following appendectomy. mBio.

[190] Fuller R. (1991). Probiotics in human medicine. Gut.

[191] Collins M.D., Gibson G.R. (1999). Probiotics, prebiotics, and synbiotics: Approaches for modulating the microbial ecology of the gut. Am. J. Clin. Nutr..

[192] Klemashevich C., Wu C., Howsmon D., Alaniz R.C., Lee K., Jayaraman A. (2014). Rational identification of diet-derived postbiotics for improving intestinal microbiota function. Curr. Opin. Biotechnol..

[193] Routy B., Le Chatelier E., Derosa L., Duong C.P.M., Alou M.T., Daillère R., Fluckiger A., Messaoudene M., Rauber C., Roberti M.P. (2018). Gut microbiome influences efficacy of PD-1-based immunotherapy against epithelial tumors. Science.

[194] Gopalakrishnan V., Helmink B.A., Spencer C.N., Reuben A., Wargo J.A. (2018). The Influence of the Gut Microbiome on Cancer, Immunity, and Cancer Immunotherapy. Cancer Cell.

[195] Wong S., Slavcev R.A. (2015). Treating cancer with infection: A review on bacterial cancer therapy. Lett. Appl. Microbiol..

[196] Quera R., Espinoza R., Estay C., Rivera D. (2014). Bacteremia as an adverse event of fecal microbiota transplantation in a patient with Crohn’s disease and recurrent Clostridium difficile infection. J. Crohn’s Colitis.

[197] Schwartz M., Gluck M., Koon S. (2013). Norovirus gastroenteritis after fecal microbiota transplantation for treatment of Clostridium difficile infection despite asymptomatic donors and lack of sick contacts. Am. J. Gastroenterol..

[198] Smith M.B., Kelly C., Alm E.J. (2014). Policy: How to regulate faecal transplants. Nature.

[199] Lev-Sagie A., Goldman-Wohl D., Cohen Y., Dori-Bachash M., Leshem A., Mor U., Strahilevitz J., Moses A.E., Shapiro H., Yagel S. (2019). Vaginal microbiome transplantation in women with intractable bacterial vaginosis. Nat. Med..

[200] Carvalho R., Vaz A., Pereira F.L., Dorella F., Aguiar E., Chatel J.M., Bermudez L., Langella P., Fernandes G., Figueiredo H. (2018). Gut microbiome modulation during treatment of mucositis with the dairy bacterium Lactococcus lactis and recombinant strain secreting human antimicrobial PAP. Sci. Rep..

[201] Park C.H., Lee A.R., Lee Y.R., Eun C.S., Lee S.K., Han D.S. (2019). Evaluation of gastric microbiome and metagenomic function in patients with intestinal metaplasia using 16S rRNA gene sequencing. Helicobacter.

[202] Iida N., Dzutsev A., Stewart C.A., Smith L., Bouladoux N., Weingarten R.A., Molina D.A., Salcedo R., Back T., Cramer S. (2013). Commensal bacteria control cancer response to therapy by modulating the tumor microenvironment. Science.

[203] Xiang S., Fruehauf J., Li C.J. (2006). Short hairpin RNA-expressing bacteria elicit RNA interference in mammals. Nat. Biotechnol..

[204] Zhang L., Gao L., Zhao L., Guo B., Ji K., Tian Y., Wang J., Yu H., Hu J., Kalvakolanu D.V. (2007). Intratumoral delivery and suppression of prostate tumor growth by attenuated Salmonella enterica serovar typhimurium carrying plasmid-based small interfering RNAs. Cancer Res..

[205] Hu B., Kou L., Li C., Zhu L.P., Fan Y.R., Wu Z.W., Wang J.J., Xu G.X. (2009). Bifidobacterium longum as a delivery system of TRAIL and endostatin cooperates with chemotherapeutic drugs to inhibit hypoxic tumor growth. Cancer Gene Ther..

[206] Loeffler M., Le’Negrate G., Krajewska M., Reed J.C. (2008). IL-18-producing Salmonella inhibit tumor growth. Cancer Gene Ther..

[207] Yoon W.S., Choi W.C., Sin J.I., Park Y.K. (2007). Antitumor therapeutic effects of Salmonella typhimurium containing Flt3 Ligand expression plasmids in melanoma-bearing mouse. Biotechnol. Lett..

[208] Nguyen V.H., Kim H.S., Ha J.M., Hong Y., Choy H.E., Min J.J. (2010). Genetically engineered Salmonella typhimurium as an imageable therapeutic probe for cancer. Cancer Res..

[209] Jia L.J., Xu H.M., Ma D.Y., Hu Q.G., Huang X.F., Jiang W.H., Li S.F., Jia K.Z., Huang Q.L., Hua Z.C. (2005). Enhanced therapeutic effect by combination of tumor-targeting Salmonella and endostatin in murine melanoma model. Cancer Biol. Ther..

[210] Li X., Fu G.F., Fan Y.R., Liu W.H., Liu X.J., Wang J.J., Xu G.X. (2003). Bifidobacterium adolescentis as a delivery system of endostatin for cancer gene therapy: Selective inhibitor of angiogenesis and hypoxic tumor growth. Cancer Gene Ther..

[211] Massa P.E., Paniccia A., Monegal A., de Marco A., Rescigno M. (2013). Salmonella engineered to express CD20-targeting antibodies and a drug-converting enzyme can eradicate human lymphomas. Blood.

[212] Dang L.H., Bettegowda C., Huso D.L., Kinzler K.W., Vogelstein B. (2001). Combination bacteriolytic therapy for the treatment of experimental tumors. Proc. Natl. Acad. Sci. USA.

[213] Zhao M., Yang M., Li X.M., Jiang P., Baranov E., Li S., Xu M., Penman S., Hoffman R.M. (2005). Tumor-targeting bacterial therapy with amino acid auxotrophs of GFP-expressing Salmonella typhimurium. Proc. Natl. Acad. Sci. USA.

[214] Blanco-Toribio A., Muyldermans S., Frankel G., Fernández L. (2010). Direct injection of functional single-domain antibodies from E. coli into human cells. PLoS ONE.

[215] Borody T.J., Eslick G.D., Clancy R.L. (2019). Fecal microbiota transplantation as a new therapy: From Clostridioides difficile infection to inflammatory bowel disease, irritable bowel syndrome, and colon cancer. Curr. Opin. Pharmacol..

